# Inorganic Nanomaterials with Intrinsic Singlet Oxygen Generation for Photodynamic Therapy

**DOI:** 10.1002/advs.202102587

**Published:** 2021-09-24

**Authors:** Muhammad Rizwan Younis, Gang He, Junle Qu, Jing Lin, Peng Huang, Xing‐Hua Xia

**Affiliations:** ^1^ Marshall Laboratory of Biomedical Engineering International Cancer Center Laboratory of Evolutionary Theranostics (LET) School of Biomedical Engineering Shenzhen University Health Science Center Shenzhen 518060 China; ^2^ Key Laboratory of Optoelectronic Devices and Systems of Ministry of Education and Guangdong Province College of Optoelectronic Engineering Shenzhen University Shenzhen 518060 China; ^3^ State Key Laboratory of Analytical Chemistry for Life Science and Collaborative Innovation Center of Chemistry for Life Sciences School of Chemistry and Chemical Engineering Nanjing University Nanjing 210093 P.R. China

**Keywords:** extinction coefficient, nano‐photosensitizers, photodynamic therapy, quantum yield, singlet oxygen

## Abstract

Inorganic nanomaterials with intrinsic singlet oxygen (^1^O_2_) generation capacity, are emerged yet dynamically developing materials as nano‐photosensitizers (NPSs) for photodynamic therapy (PDT). Compared to previously reported nanomaterials that have been used as either carriers to load organic PSs or energy donors to excite the attached organic PSs through a Foster resonance energy transfer process, these NPSs possess intrinsic ^1^O_2_ generation capacity with extremely high ^1^O_2_ quantum yield (e.g., 1.56, 1.3, 1.26, and 1.09) than any classical organic PS reported to date, and thus are facilitating to make a revolution in PDT. In this review, the recent advances in the development of various inorganic nanomaterials as NPSs, including metal‐based (gold, silver, and tungsten), metal oxide‐based (titanium dioxide, tungsten oxide, and bismuth oxyhalide), metal sulfide‐based (copper and molybdenum sulfide), carbon‐based (graphene, fullerene, and graphitic carbon nitride), phosphorus‐based, and others (hybrids and MXenes‐based NPSs) are summarized, with an emphasis on the design principle and ^1^O_2_ generation mechanism, and the photodynamic therapeutic performance against different types of cancers. Finally, the current challenges and an outlook of future research are also discussed. This review may provide a comprehensive account capable of explaining recent progress as well as future research of this emerging paradigm.

## Graphical abstract

Inorganic nano‐photosensitizers possess higher extinction coefficient, resistance to photobleaching, larger absorption cross‐section, and high singlet oxygen quantum yield than their organic counterparts. These fascinating physiochemical features endow them with promising potential for reactive oxygen species‐mediated photodynamic tumor cell killing, antibacterial therapy, thermal ablation‐induced antitumor photothermal therapy, and simultaneous tumor diagnosis and treatment for multimodal cancer theranostics.

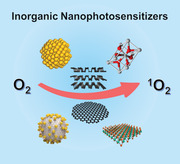

## 1 Introduction

Photodynamic therapy (PDT) has emerged as an alternative tumor ablation approach with high spatio‐temporal precision and reduced long‐term morbidity.^[^ ^1^, ^2^ ^]^ In principle, PDT involves the utilization of three basic components: light, molecular oxygen (O_2_), and a photosensitizer (PS)^[^ ^3^, ^4^ ^]^ to generate highly cytotoxic reactive oxygen species (ROS) to kill cancer cells through either type‐I or type‐II photochemical reaction mechanism.^[^ ^5^, ^6^ ^]^ In type‐I mechanism, the excited triplet state PS directly interacts with adjacent biomolecules in cancer cells and generates radical cations or anions, which can subsequently interact with oxygen (O_2_) to produce ROSs like superoxide anion (O_2_^·−^), hydroxyl radical (·OH), hydrogen peroxide (H_2_O_2_), etc.^[^ ^7^, ^8^ ^]^ In the type‐II mechanism, the excited triplet state PS directly sensitizes O_2_ (the ground state is a triplet) and generates highly cytotoxic ^1^O_2_ inside cancer cells.^[^ ^9^ ^]^ Generally, cytotoxic ^1^O_2_ has been recognized as one of the ROS responsive for PDT,^[^ ^10^ ^]^ which can kill cancer cells through multifarious pathways, such as induction of cell apoptosis or necrosis, stimulation of robust inflammatory immune response, and disruption of tumor microvasculature.^[^ ^3^, ^5^ ^]^ Though PDT has shown quite appreciable performance in treating certain cancers (e.g., skin cancers, oral cancers),^[^ ^11^ ^]^ it is still very challenging to serve as a first‐line therapeutic modality for cancer in clinics due to the existing certain limitations, such as lack of an ideal PS with high tumor selectivity and ROS generation efficacy, lack of efficient methods to select right light dosimetry for PDT, and the lack of approaches for effective monitoring of treatment response. Among them, the lack of an ideal PS with desired properties is currently the key hurdle in the advancement and clinical applications of PDT. Though a variety of small organic PSs including porphyrin structures, synthetic dyes, and natural products have been used in clinics, most of them can only show limited PDT efficacy against cancers due to their inherent features, including poor water solubility, insufficient photostability, low extinction coefficient, low absorption in the near‐infrared (NIR) region, insufficient ^1^O_2_ quantum yield, and poor cancer selectivity.^[^ ^8^, ^12^ ^]^ Therefore, it is highly desirable to develop new PSs capable of overcoming the limitations of currently used organic PSs, thus augmenting therapeutic outcomes against cancers.

Over the past few decades, the advancement in nanotechnology has offered an alternative approach to refine the performance of current PSs and overcome some of the challenges for cancer PDT. A variety of nanomaterials, including biocompatible polymer‐, metal‐, carbon material‐, silica‐, and semiconductor‐based nanomaterials have been actively developed as PSs for cancer PDT. Compared to classic organic PSs, the utility of these nanomaterials as inorganic nano‐photosensitizers (NPSs) for cancer PDT has many advantages: 1) large extinction coefficients capable of instructing an efficient energy transfer process for photosensitization; 2) facile surface modification capable of conjugating target ligands and functional groups to improve tumor selectivity; 3) large surface‐to‐volume ratios due to their nanoscale size at least 1D within 100 nm; 4) high ability to take the enhanced permeability and retention (EPR) effect, capable of increasing accumulation in solid tumor tissues; 5) high ability to integrate PSs with chemodrugs and imaging modalities capable of achieving image‐guided treatment against cancers. Generally, most of these nanomaterials utilized in PDT can serve as either carrier to load organic PSs or energy transducers to excite the attached PSs through an energy transfer process. Alternatively, recent studies have also shown that certain nanomaterials with unique optical properties can be designed as direct PSs with intrinsic ^1^O_2_ generation capacity, thereby they can produce ^1^O_2_ by themselves upon light irradiation. The direct generation of ^1^O_2_ from these inorganic nanomaterials without incorporating traditional organic PSs can effectively achieve high ^1^O_2_ quantum yield as they are highly resistant to photobleaching and possess a large extinction coefficient.^[^ ^13^, ^14^, ^15^ ^]^ Moreover, some of these nanomaterials can offer a different sensitizing mechanism to produce ^1^O_2_ as compared to that of carriers or energy transducers. As such, the development of inorganic nanomaterials as NPSs has attracted substantial research scrutiny among the scientific community and become a research hotspot, which can open promising avenues for the refinement of ideal NPSs for efficient PDT against cancers.

**Scheme 1:**
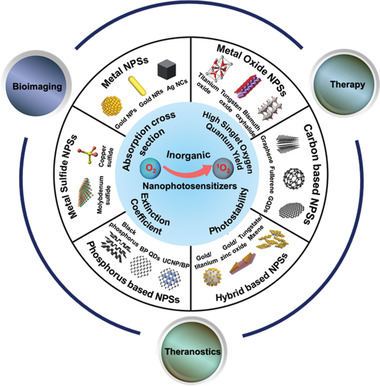
Representation of a wide variety of inorganic NPSs with intrinsic ^1^O_2_ generation capacity for PDT as well as their potential for tumor imaging, therapy, and multimodal imaging guided synergistic therapy.

In this review, we summarize the recent advances in the development of various inorganic nanomaterials as direct ^1^O_2_ generative NPSs for cancer PDT. We exclusively focused on inorganic nanomaterial‐based NPSs reported to date, including metal‐based NPSs (gold, silver, and tungsten‐based NPSs), metal oxide‐based NPSs (titanium dioxide, tungsten oxide, and bismuth oxyhalide‐based NPSs), metal sulfide‐based NPSs (copper and molybdenum sulfide‐based NPSs), carbon‐based NPSs (graphene and graphitic carbon nitride‐based NPSs), phosphorus‐based NPSs, and others (hybrid‐based NPSs and MXenes‐based NPSs) (**Scheme** **1**).

A summary of these NPSs is provided in **Table** **1**, and the reported ^1^O_2_ quantum yield for each NPS (where available) is also listed. We also critically elaborate the ^1^O_2_ generation mechanism and the design principle of these newly emerged inorganic NPSs that endowed them to achieve a high ^1^O_2_ quantum yield. In the end, the existing challenges as well as future prospects are also discussed, which will assist and guide the material chemists to develop new nanomaterials as efficient NPSs for improving cancer PDT. Though many excellent and state of the art reviews covering a range of topics related to PDT have been previously published even from our group,^[^ ^4^, ^16^ ^]^ the most of them focused on the discussion of the role of nanomaterials as 1) carriers to load organic PSs, 2) donors to excite the attached PSs (e.g., upconversion nanoparticle‐based PSs), or 3) photothermal agents.^[^ ^1^, ^17^, ^18^ ^]^ Until now, there is still no review paper to comprehensively discuss the role of inorganic nanomaterials as direct ^1^O_2_ generative materials for PDT. We hope that this first in‐depth rigorous review regarding ^1^O_2_ generative NPSs can fully and strictly address the topic of advanced materials, providing recent progress as well as an outlook of future research of this emerging paradigm. We believed that this comprehensive account will provide sufficient guidance to biomaterial scientists and biological researchers to develop highly efficient next‐generation inorganic PDT agents, as well as explore their therapeutic potential and toxicity in detail to accelerate their translation into clinics.

**Table 1:** Summary of inorganic nanomaterial‐based NPSs reported till to date for ROS generation mediated cancer PDT

Class	Material	Irradiation Source	*λ*_max_	Quantum Yield	Tumor model	Injection mode/dose	Application	Ref.
Metal‐based NPs	Au NPs	100 W Hg lamp, pulsed laser	514, 532	0.037, 0.07	–	–	Mechanism exploration of ^1^O_2_ generation	[32, 33]
	Ag NPs	100 W Hg lamp	530	0.155	–	–	Mechanism exploration of ^1^O_2_ generation	[32]
	Pt NPs	100 W Hg lamp	508	0.085	–	–	Mechanism exploration of ^1^O_2_ generation	[32]
	Au‐Ag shell satellite	Circular polarized light (6 mW cm^−2^)	532	1.09	HeLa tumor	i.v. injection (100 nm)	Bimodal CT/PAI‐guided PDT	[34]
	Au NRs	Two‐photon laser (300 W cm^−2^), LED light (130 mW cm^−2^)	765, 808, 835, 940	–	HeLa cells B16F0 melanoma tumor	i.v. injection (100 µg mL^−1^)	Two‐photon imaging and in vitro PDT, In vivo PDT/PTT	[35, 37]
	Au NEs	LED light, 130 mW cm^−2^	915, 1064	0.19, 0.22	B16F0 tumor	Intratumoral injection (10 mg mL^−1^)	In vivo PDT/PTT	[38]
	Au NCs	980 nm laser (0.5 W cm^−2^)	380	0.048	HeLa cells, zebrafish	Microinjection (100 µg mL^−1^)	In vivo FL imaging, gene delivery, in vitro PDT	[45]
	Ag NCs	White light (150 mW cm^−2^)	480	1.26	MCF‐7	50–500 µm	FL imaging, in vitro PDT	[48]
Metal oxide‐based NPSs	TiO_2_ NPs	Pulsed laser	400	0.2	–	–	Mechanism exploration of ^1^O_2_ generation	[53]
	W_18_O_49_ NWs	CW NIR laser (200 mW cm^−2^), (1.2 W cm^−2^), 6 Gy for RT	980	0.29	B16F0 tumor, 4T1 tumor	Intratumoral injection (15 mg kg^−1^), Intratumoral injection (1.4 mg mL^−1^)	In vivo PDT/PTT, CT imaging‐guided multimodal (PDT/PTT/RT) therapy	[65, 66]
	WO NPs	CW NIR II laser (2 W cm^−2^)	–		HeLa tumor	Intratumoral injection (1 mg mL^−1^)	In vivo PAI‐guided PDT/PTT	[125]
	BiOCl NSs	Mercury lamp (300 W cm^−2^)	350	–	MCF‐7	(0–200 µg mL^−1^)	In vitro PDT	[68]
	UCNP/BiOCl	CW laser (0.5 W cm^−2^)	300	–	U14 murine cervical carcinoma	i.v. injection (1 mg mL^−1^)	Upconversion luminescence (UCL) and in vivo PDT	[69]
	BiOBr: Yb/Tm	CW laser (0.9 W cm^−2^)	350	–	U14 murine cervical carcinoma	Intratumoral injection (1 mg mL^−1^)	UCL/CT imaging‐guided in vivo PDT	[70]
Carbon‐based NPSs	C60–PDA–rGO	Xe lamp (50 W cm^−2^)	–		HeLa cells	(50 µg mL^−1^)	In vitro PDT/PTT	[85]
	GQDs	CW laser (1 W cm^−2^), two photon laser (2.64 mW cm^−2^)	300	–	U251 glioma cells, Bacterial pathogens	200 µg mL^−1^, 0–1 µgmL^−1^	In vitro PDT, TP imaging and antibacterial PDT	[87, 89]
	N‐GQDs, Amino N‐GQDs	CW (0.1 W cm^−2^), Two photon laser (2.3936 mW cm^−2^)	–	0.60, 0.53	Bacterial pathogens	0–10 µg mL^−1^	In vitro antibacterial PDT	[90]
	GQDs	White light (80 mW cm^−2^)	532	1.3	MDA MB‐231 tumor	Intratumoral injection (4 mg kg^−1^)	In vivo bimodal FL/PAI‐guided PDT/PTT	[14]
	Au NRs@SiO_2_‐CDs	CW laser (0.1 W cm^−2^ for PDT and 0.5 W cm^−2^ for PTT)	808	1.3	B16F0 tumor	i.v. injection (2 mg mL^−1^)	In vivo bimodal FL/PAI‐guided PDT/PTT	[92]
	UCNP‐GQDs	CW laser, 980 nm laser (0.5 W cm^−2^)	300	1.5	4T1 tumor	i.v. injection	In vivo FL imaging‐guided PDT	[93]
	Gd@GCNs	LED light (100 mW cm^−2^)	550	0.51	SCC‐7 tumor	i. v. injection, (0.1 mmol Gd kg^−1^)	In vivo bimodal FL/MRI‐guided PDT	[94]
	C_3_N_4_ NSs	LED light (20 mW cm^−2^)	300	–	HeLa cells	(50 µg mL^−1^)	In vitro PDT	[98]
	g‐C_3_N_4_ nanospheres	Halogen lamp (300 W cm^−2^)	350	–	MDA‐MB‐231 cells	(5–25 µg mL^−1^)	In vitro PDT/chemotherapy	[100]
	UCNP/C_3_N_4_ NSs	CW laser (2.5 W cm^−2^)	350	–	H22 tumor	i.v. injection (1 mg mL^−1^)	In vivo PDT	[103]
	UCNP/C_3_N_4_ QDs	CW laser (2.5 W cm^−2^), (1.5 W cm^−2^)	350, 370	–	OEC‐M1 cells, CAL 27 tumor	(250 µg mL^−1^)	In vitro PDT, In vivo PDT	[104, 105]
Metal Sulfide‐based NPSs	MoS_2_ QDs	CW laser	630	–	–	(100 µg mL^−1^)	^1^O_2_ detection in solution	[73]
	CuS NCs	CW NIR laser (0.6 W cm^−2^)	808	–	B16 tumor	Intratumoral injection (15 mg kg^−1^)	In vivo PDT/PTT	[74]
Phosphorus‐based NPSs	BP NSs	CW laser (0.5 W cm^−2^)	450	0.91	MDA‐MB‐231 tumor	Intratumoral injection (500 µg mL^−1^)	In vivo PDT	[107]
	UCNP/BP NSs	CW laser (1.44 W cm^−2^)	–	–	U14 tumor	i.v. injection (1 mg mL^−1^)	In vivo PDT	[108]
	BP QDs	CW laser (80 W cm^−2^ for PDT and 2 W cm^−2^ for PTT)	–	0.74	4T1 tumor	Intratumoral injection (500 µg mL^−1^)	In vivo PDT/PTT	[111]
Hybrid‐based NPSs	Au NRs/TiO_2_	CW NIR I laser	808	–	–	–	Mechanism exploration of ^1^O_2_ generation	[24]
	Au NPs/TiO_2_	Simulated sunlight, 532 nm laser	532	2 × 10^−6^	–	–	Mechanism exploration of ^1^O_2_ generation	[25]
	Au NCs/TiO_2_	650 nm laser (0.5W cm^−2^)	650	–	U14 tumor	i.v. injection (500 µg mL^−1^)	In vivo PDT	[27]
	Au NPs/ZnO	Simulated sunlight	400	–	–	–	In vitro antibacterial PDT	[116]
	Cs_x_WO_3_ NRs	CW NIR (I, II) laser (2 W cm^−2^)	808	–	HeLa tumor	Intratumoral injection (1 mg mL^−1^)	In vivo bimodal CT/PAI‐guided PDT/PTT	[121]
	W_2_C NPs	CW NIR II laser (0.8 W cm^−2^)	1064	–	S180 tumor	i.v. injection (10 mg kg^−1^)	In vivo bimodal CT/PAI‐guided PDT/PTT	[122]
	SnWO_4_ NPs	LED light (0.2 W cm^−2^)	465	–	4T1 tumor	Intraperitoneal injection (23 mg kg^−1^)	In vivo PDT	[123]
	UCNPs@g‐C_3_N_4_‐Au_25_	CW laser (2.5 W cm^−2^)	–	0.74	H22 tumor	i.v. injection (400 µg mL^−1^)	In vivo trimodal CT/FL/MRI‐guided PDT/PTT	[119]

## 2 Design Principle of Inorganic NPSs and ^1^O_2_ Generation Mechanism

**Figure 1:**
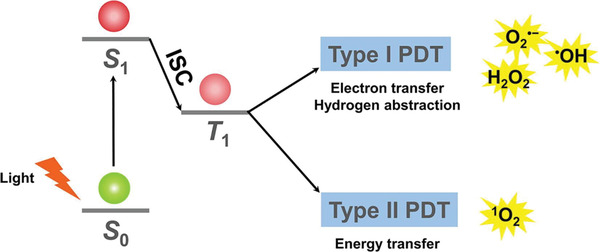
Demonstration of the chemistry behind the type‐I and type‐II photodynamic therapy and the generation of ^1^O_2_ and other reactive oxygen species (ROSs), respectively. Reproduced with permission.^[^ ^19^ ^]^ Copyright 2019, Elsevier.

Under photo‐excitation, the most of inorganic NPSs shifted from ground energy state (S_0_) to the singlet excited energy state (S_1_). In nanoseconds, they moved from S_1_ to a triplet energy state (T_1_) via intersystem crossing, followed by decay back to S_0_ by releasing the absorbed photon energy to the surrounding or surface adsorbed molecular oxygen (^3^O_2_) to generate ^1^O_2_. This mechanism is based on the type‐II pathway of ^1^O_2_ generation. **Figure** **1** demonstrates ^1^O_2_ generation by Type‐II PDT pathway along with the generation of other ROSs by Type‐I pathway.^[^ ^19^ ^]^ Notably, the structural design of inorganic NPSs played a decisive role in ^1^O_2_ generation. It has been demonstrated that molecular O_2_ could selectively binds on a specific crystalline facet of nanomaterial, leading to morphology‐dependent production of ^1^O_2_.^[^ ^13^ ^]^ For example, oxygen exists as a molecular form when bind on the (111) crystalline facet of Ag NPs, but presents as an atomic form on both (100) and (110), thereby, ^1^O_2_ can only be formed on Ag NPs that possess Ag (111) surface. In addition, the size of inorganic NPSs also had a profound effect on ^1^O_2_ generation. In general, the smaller size inorganic NPSs produced a high ^1^O_2_ quantum yield as more amounts of O_2_ were adsorbed on the surface of smaller NPs due to the high surface area to volume ratio. Whereas, for plasmonic NPs, the effect of size could be more complex, as plasmonic NPs with a bigger size generated more hot electrons compared to that of a smaller size, thus producing more ^1^O_2_.^[^ ^20^ ^]^ Surface modification of inorganic NPSs also played an important role in improving their ROS generation capacity. The chemical reduction of graphene oxide quantum dots (rGOQDs) significantly lower the bandgap and valence band (VB), which led to generating more electron–hole pairs, resulting in enhanced ROS generation by rGOQDs than GOQDs under white light irradiation.^[^ ^21^ ^]^ In addition, the coupling of two different inorganic NPs to develop hybrid‐based inorganic NPSs is also an effective strategy to improve ROS yield due to slower electron–hole pairs recombination.^[^ ^22^ ^]^ The fabrication process is also an important factor that endows inorganic NPSs with a high ^1^O_2_ quantum yield. For example, the hydrothermal treatment of polythiophene engineers the energy levels of the resultant graphene QDs (GQDs), which lead to a huge energy gap of 49.3 kcal mol^−1^ between the ground state (S0) and excited singlet state (S1) of GQDs. Given the fact that the formation of ^1^O_2_ from ^3^O_2_ requires an amount of 22.5 kcal mol^−1^ energy, GQDs offered high ^1^O_2_ quantum yield of 1.3 due to multistate sensitization ^1^O_2_ generation mechanism, as the ^3^O_2_ received an equal amount of energy on each transition either from S1 to T1 or from T1 to S0 to be converted into ^1^O_2_, resulting in high ^1^O_2_ generation.^[^ ^14^ ^]^ In addition, strong acid oxidation of precursor material to fabricate GQDs results in GQDs with an ample amount of surface oxygenated functional groups, promoting an efficient O_2_ quenching of triplet states, which leads to enhance ^1^O_2_ quantum yield (1.56).^[^ ^23^ ^]^

**Figure 2:**
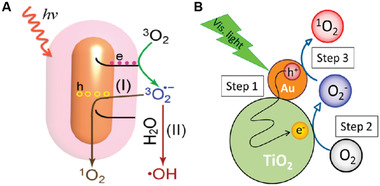
Representation of ^1^O_2_ generation by inorganic hybrid‐based NPSs following type‐I PDT pathway. Transfer of an electron from A) AuNRs and B) AuNPs to TiO_2_ shell, resulting in reduced charge recombination and ^1^O_2_ generation by Type‐I pathway. (A) Reproduced with permission.^[^ ^24^ ^]^ Copyright 2014, Royal Society of Chemistry. (B) Reproduced with permission.^[^ ^25^ ^]^ Copyright 2014, American Chemical Society.

In contrast to the type‐II ^1^O_2_ generation pathway, inorganic semiconductor NPSs and hybrid‐based NPSs follow the type‐I pathway of ^1^O_2_ generation. Briefly, O_2_ absorbed on the surface of inorganic NPSs especially TiO_2_ is reduced to superoxide anion (O_2_^·−^) by an electron in the conduction band (CB), which was then oxidized by the hole in the valence band (VB) to form ^1^O_2_ (**Figure** **2**).^[^ ^24^, ^25^ ^]^ It is noteworthy to mention that the hydrodynamic size and the band edge structures of inorganic metal‐oxide NPSs are very crucial in the generation of ROS. Compared to their bulk counterparts, the smaller size NPSs generated more ROS under UV irradiation, presumably because the NPs had larger surface areas capable of providing more reaction sites for the absorption of UV light. Second, as ROS generation is dictated by the photoexcitation‐driven interfacial electron transfer process, only the metal‐oxide NPSs with band gap energy (Eg) smaller than the incident photon energy (i.e., ≈3.4 eV for the 365 nm UV) can be photoexcited to generate electrons in CB and holes in the VB, which subsequently react with an aqueous electron acceptor (e.g., molecular oxygen) and donor (e.g., H_2_O and OH^−^), respectively, ultimately producing different types of ROS.^[^ ^26^ ^]^

Though hybrid‐based inorganic NPSs followed the same ^1^O_2_ generation mechanism as metal‐oxide NPSs, they demonstrated an enhanced ^1^O_2_ generation capacity due to reduced electron–hole pairs recombination than metal‐oxide NPs alone, as electron could be transferred from semiconductor to the metal NPs or vice versa depending upon the excitation wavelength and Schottky barrier. Notably, the deposited metal NPs onto semiconductor NPs alter the electrical distribution of the hybrid, which hampered the recombination process. However, the size of semiconductor NPs, as well as the amount of loaded metal NPs, should be considered because larger size semiconductor NPs significantly slow down the recombination process than smaller size NPs, resulting in improved ROS generation. Whereas, the higher amount of metal NPs would impede the adsorption of O_2_ molecules on the surface of semiconductor NPs_,_ leading to low ^1^O_2_ generation. We hope that this thorough and balanced discussion would greatly assist and guide the development of next‐generation inorganic NPSs with a high ^1^O_2_ quantum yield.^[^ ^27^ ^]^

## 3 Metal‐Based NPSs

In recent years, noble metal nanomaterials (NMs) have gained significant attention due to their unique physiochemical and optical properties, which have found wide applications in industry, environment, and health.^[^ ^28^ ^]^ Among these noble metal NMs, gold (Au) and silver (Ag) NMs have been of considerable research interest.^[^ ^29^ ^]^ For example, Au NMs with diverse morphologies such as nanoparticles (Au NPs), nanorods (Au NRs), nanoclusters (Au NCs), nanoshells, nanoechinus (Au NE), and nanostars (Au NSs) have been actively developed. These Au NMs possess many versatile properties, such as high chemical inertness, easily tunable optical properties, high biocompatibility, large extinction coefficients and facile surface modifications.^[^ ^30^ ^]^ More importantly, some well‐designed AuNMs also possess strong localized surface plasmon resonance (LSPR) properties, which allowed them to efficiently absorb light and convert it into heat to trigger photothermal effect.^[^ ^31^ ^]^ In addition, recent studies have also revealed that AuNMs could be directly sensitized to generate highly cytoxicic ^1^O_2_, which make them potential candidate as an additional weapon in PDT capable of providing a synergistic effect of photothermal therapy (PTT) with PDT to improve therapeutic efficacy. Thus, AuNMs have found tremendous biomedical applications from diagnosis to therapy.

### 3.1 Gold‐Based NPSs

**Figure 3:**
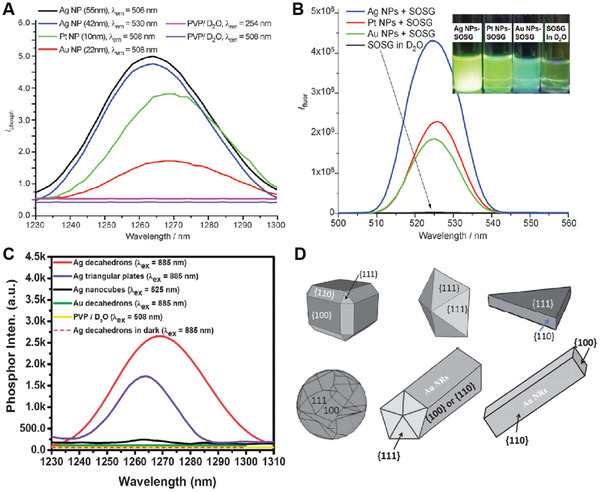
A) Phosphorescence emission spectra of ^1^O_2_ sensitized by Au, Ag, and Pt NPs in D_2_O. B) Fluorescence spectra of SOSG in D_2_O in the presence and absence of any metal NPs. The inset shows green fluorescence emission from SOSG in four different D_2_O solutions in the presence and absence of metal NPs. Reproduced with permission.^[^ ^32^ ^]^ Copyright 2011, Wiley‐VCH. C) Fluorescence spectra of SOSG aqueous solutions in the presence of 1 mg of different metal NPs. D) Correlation of morphology and index number of different crystalline surfaces for metal NPs (such as Ag and Au) having a fcc crystalline structure. Reproduced with permission.^[^ ^13^ ^]^ Copyright 2013, Royal Society of Chemistry.

In 2011, Raviraj et al. first reported an unprecedented observation that noble metal NPs could be directly sensitized to produce ^1^O_2_ without using organic photosensitizers.^[^ ^32^ ^]^ In this study, three different noble metal NPs, including Au NPs (*d* = 22 nm), Ag NPs (*d* = 42 and 55 nm) and Pt NPs (*d* = 10 nm) were synthesized. All these NPs exhibited one major strong LSPR band around 398–530 nm. Upon irradiation at the LSPR band of each NPs, the production of ^1^O_2_ was monitored by the direct observation of ^1^O_2_ phosphorescence at 1264 and 1268 nm (**Figure** **3**A). The subsequent measurement of ^1^O_2_ quantum yield using singlet oxygen sensor green (SOSG) as a fluorescent ^1^O_2_ indicator demonstrated that Ag NPs possessed a higher value (0.155) as compared to Pt NPs (0.085) or Au NPs (0.037) (Figure 3B). It was plausible that the energy transfer from LSPR of these metal NPs to molecular oxygen might be a possible reason to produce ^1^O_2_ upon light irradiation. Based on these results, they subsequently revealed that the generation of ^1^O_2_ was highly dependent on the morphologies of Au and Ag NPs.^[^ ^13^ ^]^ They demonstrated that Ag decahedrons and Au triangular plates could produce strong ^1^O_2_ phosphorescence emission at ≈1263 nm upon irradiation at 885 nm, while Ag nanocubes and Au decahedrons only produced negligible amount of ^1^O_2_ (Figure 3C). Moreover, strong ^1^O_2_ phosphorescence emission was also observed from penta‐twinned Au NRs upon irradiation at a longitudinal LSPR band (≈885 nm), while little ^1^O_2_ was formed when exciting at the transverse LSPR band. Mechanism studies showed that the morphology‐dependent production of ^1^O_2_ from these nanomaterials was due to the selective binding of molecular O_2_ on a specific crystalline facet (Figure 3D). For example, oxygen exists as a molecular form when binding on the Ag (111) surface but presents as an atomic form on both Ag (100) and Ag (110) surface, thereby, ^1^O_2_ can only be formed on Ag NPs that possess Ag (111) surface like Ag decahedrons and Ag triangular plates.

In 2013, Pasparakis et al. studied the effect of different laser sources on the generation of ^1^O_2_ from Au NPs by using continuous wave (CW) laser and pulsed laser sources.^[^ ^33^ ^]^ Citrate stabilized Au NPs with a mean diameter of 40 nm and an LSPR band at 524 nm were prepared. The generation of ^1^O_2_ was confirmed by direct observation of ^1^O_2_ phosphorescence at 1270 nm and a gradual reduction in the absorption of 1,3‐diphenylisobenzofuran (DPBF), a ^1^O_2_ indicator. They found that the ^1^O_2_ generation efficiency upon irradiation with pulsed laser (7 ns, 532 nm) was about 0.07, which was more than twofold higher than that with CW laser (0.03) at 532 nm. The difference in ^1^O_2_ generation efficacy was presumably due to two different pathways involved in sensitizing O_2_ during the irradiation of Au NPs. When irradiated under a CW laser, the ^1^O_2_ was generated mainly through a plasmon and hot‐electron emission mechanism. However, when irradiated under a pulsed laser, a combination of the plasmon‐activated pathway and indirect photothermal pathway that potentially induces particle fragmentation and increases thermionic electron emission contributed largely to ^1^O_2_ production. This study demonstrated that irradiation of Au NPs with a pulse laser was more efficient to trigger ^1^O_2_ production compared to CW laser, which subsequently led to much higher cancer cell death. Afterward, Chadwick et al. confirmed that irradiation of Au NPs with a pulsed laser can proceed through the equilibrated hot electrons with temperature reached to several thousand degrees. In addition, they also found that the size of Au NPs had a profound effect on ^1^O_2_ generation.^[^ ^20^ ^]^ Larger Au NPs (46 nm) produced more ^1^O_2_ as compared to smaller Au NPs (15 nm). This is because that Au NPs with a bigger size generated more hot electrons as compared to that of a smaller size.

**Figure 4:**
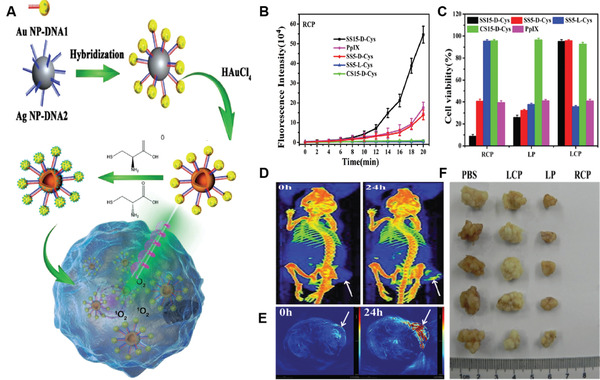
A) The illustration of self‐assembled SS NS as a chiral PDT agent. B) SOSG fluorescence intensity at 526 nm of different samples during the 532 nm irradiation under RCP light (6 mW cm^−2^). C) The HeLa cell viability incubating with different core‐shell and SS NSs after 532 nm RCP, LP and LCP light irradiation. D) CT imaging and E) PA imaging of HeLa tumor‐bearing mice taken at different time points after injection with agent (SS15‐d‐Cys). F) Tumor dissection photographs of tumor sections after various treatments. Reproduced with permission.^[^ ^34^ ^]^ Copyright 2016, Wiley‐VCH.

In 2017, Gao et al. reported an intriguing study of fabricating DNA‐driven shell‐satellite (SS) Au‐Ag plasmonic nanoparticles assemblies as chiral NPSs for cancer PDT.^[^ ^34^ ^]^ The chiral SS nanoassemblies were prepared by hybridization of complementary DNA sequences modified Au NPs and Ag NPs, followed by galvanic replacement reaction and surface coupling with cysteine enantiomers (**Figure** **4**A).

The as‐prepared chiral nanoassemblies displayed strong chiroplasmonic activities in the visible region. Importantly, upon right circular polarized (RCP) light illumination of d‐cysteine modified SS nanoassemblies that contained Au NPs with a mean size of ≈15 nm (SS15‐d‐Cys), very high ROS generation efficiency was observed. The ^1^O_2_ quantum yield was estimated to be 1.09, which was more than four times higher than that of protoporphyrin IX (PpIX) (0.23). Interestingly, irradiation of d‐cysteine modified SS nanoassemblies with Au NPs at a size of ≈5 nm (SS5‐d‐Cys) only produced negligible ROS, indicating that the structure of the nanoassemblies also played an important role in the production of ROS (Figure 4B). Attributed to the high ^1^O_2_ quantum yield of SS15‐d‐Cys, significant destruction of various tumor cells, including HeLa, MCF‐7, HepG2, and Caco‐2 tumor cells, was achieved under irradiation of SS15‐loaded cells with an RCP light (6 mW cm^−2^, 20 min) (Figure 4C). Meanwhile, SS15‐d‐Cys also showed dual‐imaging potential as the strong signal was observed at 24‐h post‐injection for both computed tomography (CT) and photoacoustic imaging (PAI), respectively. In vivo studies on subcutaneous tumor‐bearing mice were subsequently carried out, which showed remarkably strong (CT) and (PAI) signal at 24 h post‐injection, respectively, and the tumor was completely ablated under RCP light irradiation (Figure 4D–F). Thus, RCP light excitable SS nanostructures are exciting NPSs, which demonstrated the promising potential of dual‐modal imaging‐guided in vivo cancer therapy.

In addition to 0D Au NPs, 1D Au NRs have also shown the ability to directly generate ^1^O_2_ by taking advantage of their tunable LSPR bands. In 2012, Zhao et al. demonstrated that polyvinylpyrrolidone (PVP) coated Au NRs could be directly sensitized to generate ^1^O_2_ under two‐photon excitation (TPE).^[^ ^35^ ^]^ In their study, Au NRs with three different aspect ratios (≈3.5, ≈4.0, and ≈4.3) were prepared, with the maximum absorbance band at 765, 808, and 835 nm, respectively. Importantly, they demonstrated that all these three Au NRs exhibited large two‐photon absorption cross‐sections, greatly facilitating the absorption of two‐photon energy for sensitizing O_2_. Upon TPE with an 808 nm femtosecond pulse laser, the photooxidation rates of 9, 10‐anthracenediyl‐bis (methylene) dimalonic acid (ABDA) in the presence of the Au NRs were much faster than that of either Rose Bengal (RB) or Indocyanine Green (ICG), revealing that the two‐photon induced ^1^O_2_ generation capacity of these Au NRs was much larger than that of RB or ICG. Cell studies showed that the PVP‐coated Au NRs could efficiently enter HeLa tumor cells and cause significant cell death (upto 85%) following irradiation with the 808 nm femtosecond laser for 15 min, suggesting that Au NRs could serve as efficient NPSs for two‐photon induced PDT. Latterly, the same group further demonstrated that two‐photon induced ^1^O_2_ generation abilities of Au nanospheres and short Au NRs (low aspect ratio) could be elevated when they were present in an aggregated form.^[^ ^36^ ^]^ As Au NPs normally existed in an aggregated form when entering into tumor cells, it would be beneficial to use the aggregated Au NPs for PDT under TPE.

**Figure 5:**
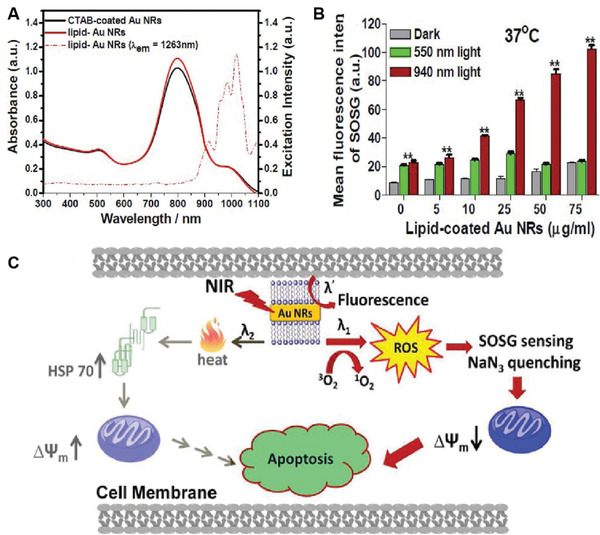
A) The UV/vis‐NIR absorption spectra (solid lines) of Au NRs as well as the excitation spectrum (dashed lines) of ^1^O_2_ phosphorescence (*λ*_em_ = 1263 nm). B) SOSG fluorescence levels determined from HeLa cells as a function of Au NRs fed at 37 °C for 4 h in dark, followed by different irradiation conditions. C) Cellular events involved in the PDT/PTT mediated cellular destruction by photo‐excited Au NRs. Reproduced with permission.^[^ ^37^ ^]^ Copyright 2016, Wiley‐VCH.

Though the TPE Au NRs and Au nanospheres have shown promising applications in PDT, the requirement of expensive femtosecond lasers and high laser doses (usually 1–48 W cm^−2^ at 808 nm) has limited their clinical applications. In 2014, Vankayala et al. demonstrated the first example of Au NRs capable of initiating efficient in vivo PDT/PTT against B16F0 melanoma tumor xenografts under single‐photon NIR laser irradiation at a very low power density (<130 mW cm^−2^).^[^ ^37^ ^]^ In their study, cationic lipid (Lipofectamine 2000) coated Au NRs with an average length and diameter of 37.3 ± 2.4 nm and 11 ± 1.2 nm, respectively, were synthesized, which had a strong NIR absorption band at ≈808 nm. They demonstrated that the ^1^O_2_ could be produced only when the Au NRs were irradiated by a long NIR light (875–1100 nm), but not by a visible or short NIR light (**Figure** **5**A). The measurement of ^1^O_2_ generation capacity in HeLa cells using the SOSG revealed a large amount of ^1^O_2_ generation upon irradiation with a 940 nm LED light, whereas little ^1^O_2_ generation was observed under 550 nm light irradiation (Figure 5B). More than ≈62% cells were dead when irradiating the Au NRs‐loaded HeLa cells (25 µg mL^−1^) by 940 nm light, which was approximately tenfold higher as compared to that irradiation at 550 nm. They demonstrated that the 940 nm irradiation‐induced ROS generation contributed mainly to cell death, which was more effective compared to the PTT effect at 550 nm. The subsequent in vivo studies in B16F0 melanoma tumor‐bearing mice showed complete destruction of tumors upon irradiation with a 915 nm NIR laser at a power density of only 130 mW cm^−2^, which was more effective compared to the Au NRs‐induced PTT effect upon irradiation by 780 nm light or chemotherapy with doxorubicin. Figure 5C illustrated the cellular events involved in the PDT/PTT mediated cellular destruction by photo‐excited Au NRs. As the Au NRs possessed excellent photostability, high resistance to enzymatic degradations, and four to six orders higher extinction coefficients than organic PSs at NIR regions, the Au NRs reported in this work could serve as promising NPSs for effective cancer PDT.

**Figure 6:**
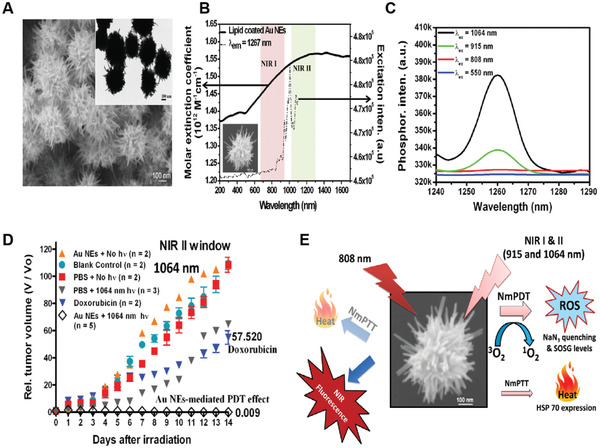
A) SEM and TEM (inset) images of the as‐synthesized Au NEs. B) UV/vis–NIR absorption spectrum (black solid line) and excitation spectrum (black dashed line) for ^1^O_2_ phosphorescence of lipid‐coated Au NEs (*λ*_em_ = 1267 nm). C) ^1^O_2_ phosphorescence emission spectra sensitized by lipid‐coated Au NEs at different excitation wavelengths. D) In vivo mediated PDT by Au NEs at NIR II window. E) Schematic representation of the working mechanisms of NIR light induced fluorescence emission as well as phototherapeutic effects exerted by Au NEs. Reproduced with permission.^[^ ^38^ ^]^ Copyright 2014, Wiley‐VCH.

To directly sensitize the formation of ^1^O_2_ for the treatment of deep‐seated tumors, the same group lately reported a unique gold nanoechinus structure (Au NE) as combinational PDT and PTT nanomaterials for ablation of tumors using NIR light in both the first and second biological windows.^[^ ^38^ ^]^ A seed‐growth procedure using double‐chain cetyltrimethylammonium bromide (DC_14_TAB) surfactant was employed to synthesize the Au NEs, with a mean hydrodynamic size of 350 ± 50 nm. Both SEM and TEM images showed that the Au NEs contained many nanorods or multiple tips on the surface of each nanoparticle (**Figure** **6**A). The Au NEs displayed a strong NIR absorption which covers both the NIR‐I (650–960 nm) and NIR‐II (1000–1300 nm) regions, presumably due to the enhanced LSPR property of the echinus‐like structure (Figure 6B). Moreover, the Au NEs also showed large molar extinction coefficients (≈0.69 × 10^12^ m ^−1^ cm^−1^ at 915 nm and ≈0.74 × 10^12^ m ^−1^ cm^−1^ at 1064 nm), which were 7–9 orders higher than traditional organic PSs and 3–4 orders higher than other reported gold nanoparticles. It was notable that the Au NEs could be excited by NIR light at either the first (915 nm) or second (1064 nm) biological windows to sensitize the formation of an obvious ^1^O_2_ phosphorescence at ≈1267 nm, consistent with the two characteristic peaks (948 and 1074 nm) found in the excitation spectrum (Figure 6C). The ^1^O_2_ quantum yields were found to be 0.19 and 0.22 at 915 nm and 1064 nm light excitation, respectively. In addition to the generation of ^1^O_2_, they also demonstrated that the Au NEs could also produce heat under irradiation with 915, 940, or 1064 nm light, thus enabling bimodal PDT and PTT to kill tumor cells. After direct injection of the Au NEs into the subcutaneously implanted B16F0 tumors in living mice, a remarkable inhibition of tumor growth was achieved when the tumors were irradiated with 915 or 1064 nm light at a power density of merely 130 mW cm^−2^, which was approximately two to three times lower than the standards set by American National Standard Institute for skin burning.^[^ ^39^ ^]^ The average tumor volume in mice at day 14 post‐treatment with 1064 nm light irradiation was found to be 0.009 of initial tumor size, which was significantly lower than that with 808 nm light (53.33) or doxorubicin (57.52) (Figure 6D).

Moreover, tumor‐bearing mice with 1064 nm light irradiation could survive for over 60 days, which was also significantly longer than that with 808 nm light (12 days) or doxorubicin (20 days). These results suggested that irradiation of Au NEs with light at NIR‐II windows to trigger both PDT and PTT effects against tumors in vivo was superior to the mere PTT effect induced by the 808 nm light (Figure 6E). More importantly, the extremely large extinction coefficients of the Au NEs along with the deeper tissue penetration depth of the NIR‐II light at 1064 nm could allow Au NEs to act as an effective agent for the treatment of deep‐located tumors in vivo.

Considering that Au nanoclusters (Au NCs) with sizes comparable to the Fermi wavelength generally possess intriguing size‐dependent optical, electronic, and chemical properties, they have recently attracted much attention for the applications in optoelectronic devices,^[^ ^40^ ^]^ catalysis,^[^ ^41^ ^]^ and fluorescent imaging.^[^ ^42^ ^]^ In 2009, Sakamoto et al. first employed single‐molecule fluorescence spectroscopy (SMS) to elucidate the photoreactivity of Au NCs.^[^ ^43^ ^]^ Au NCs with different sizes Au*_n_* (*n*, <12 or 17) and Au*_m_* (*m*, 19 or 21) were prepared via UV light irradiation of a poly(vinyl acetate) film containing HAuCl_4_ and a radical precursor. SMS images showed that the fluorescence of Au*_n_* (*n*, <12 or 17) was significantly quenched by O_2_ through an electron transfer, while the fluorescence of Au*_m_* (*m*, 19 or 21) increased greatly with increasing O_2_ concentration from 0.3% to 95%, suggesting that an increase in atom number caused a remarkable change in the photoreactivity. The increase in fluorescence intensity of Au*_m_* with the O_2_ concentration could be due to the depopulation of the triplet state through energy transfer to O_2_ molecules, which was confirmed by the appearance of ^1^O_2_ phosphorescence signal at 1270 nm. These observations suggested that the cluster size as well as spin multiplicity have profound effects on the photochemical reactivity of Au NCs, which was helpful to guide the design of noble metal clusters with tunable photochemical properties for PDT. Encouraged by this, Das et al. elucidated that the orientation of O_2_ molecules adsorbed on the surface of Au NCs with a different size was the key factor to influence the fluorescence intensity.^[^ ^44^ ^]^ Bovine serum albumin (BSA) stabilized Au NCs with a small size (Au_8_) had a superoxo type of O_2_ orientation that could enhance the fluorescence emission and induce the formation of ^1^O_2_ in the presence of O_2_. In contrast, the BSA stabilized Au NCs with a larger size (Au_25_) had a peroxo type of O_2_ orientation that showed quenched fluorescence.

**Figure 7:**
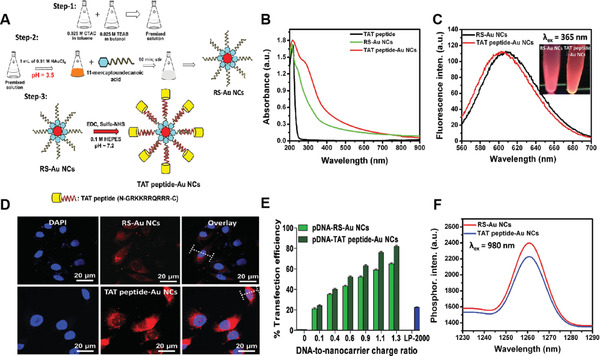
A) Schematic representation for the synthesis of red emitting RS–Au NCs and TAT peptide–Au NCs, respectively. B,C) UV/vis–NIR absorption and PL emission spectra for red fluorescent RS–Au NCs and TAT peptide–Au NCs. D) Confocal fluorescence images for photoexcitation of internalized red emitting RS–Au NCs and TAT peptide–Au NCs in HeLa cells by 533 nm excitation light and fluorescence emission was monitored from the red channel (*λ*_em_ = 570–630 nm). E) The percentage of gene transfection efficiency of pDNA‐RS‐Au NCs and p‐DNA‐TAT peptide Au NCs complexes using flow cytometry. F) ^1^O_2_ phosphorescence emission spectra of RS–Au NCs and TAT peptide–Au NCs at 980 nm excitation wavelength. Reproduced with permission.^[^ ^45^ ^]^ Copyright 2015, Wiley‐VCH.

In 2015, Hwang's group reported nucleus‐targeting Au NCs for simultaneous fluorescence imaging, gene delivery, and PDT of tumors upon NIR light irradiation.^[^ ^45^ ^]^ In their study, 11‐mercaptoundecanoic acid stabilized Au NCs (RS‐Au NCs) were first synthesized, which were then coupled with the TAT peptide (N‐GRKKRRQRRR‐C) to afford the nucleus‐targeting Au NCs (TAT‐Au NCs) with a mean particle size of 3–5 nm and molecular weight of 2100 (**Figure** **7**A). Both RS‐Au NCs and TAT‐Au NCs showed absorption in the entire spectral range (up to 900 nm) and bright red fluorescence (≈600 nm) under UV exposure (Figure 7B,C), which could allow fluorescence monitoring of the uptake of Au NCs into live tumor cells. Confocal fluorescence imaging revealed that TAT‐Au NCs exerted a higher accumulation inside the nucleus relative to that of RS‐Au NCs, indicating that the presence of TAT on the surface of Au NCs could trigger efficient delivery into the nucleus (Figure 7D). Furthermore, the TAT‐Au NCs also showed an ultrahigh gene transfection efficiency of ≈81% in HeLa cells, which was ≈3.2‐fold higher than that using LP2000, a commonly used gene carrier. The efficient transfection of the GFP gene in zebrafish using the pDNA‐TAT Au NCs complexes was also realized (Figure 7E), suggesting that the TAT Au NCs could act as very effective gene carriers. They further revealed that both RS‐Au NCs and TAT‐Au NCs could directly sensitize the formation of ^1^O_2_ under irradiation at 980 nm light (Figure 7F), with ^1^O_2_ quantum yields of 0.048 and 0.046 for RS‐Au NCs and TAT‐Au NCs, respectively. The NIR light‐activated formation of ^1^O_2_ could obviously elevate the intracellular ROS levels, thus causing significant DNA damage and inducing irreversible cell death. It was also found that the TAT‐Au NCs could induce more cell death relative to the nucleus‐nontargeting RS‐Au NCs under 980 nm light irradiation, revealing that the delivery of PSs inside the nucleus could trigger improved PDT effects to kill tumor cells. Two similar studies, which used thiolated Au NCs (Au_25_(SR)_18_)^[^ ^46^ ^]^ and captopril‐protected Au NCs (Au_25_(Cap)_18_)^[^ ^47^ ^]^ capable of emitting red fluorescence and directly sensitizing the formation of ^1^O_2_ to kill cancer cells and microbacterial cells, respectively, have also been reported, supporting the high capacity of Au NCs for both fluorescence imaging and PDT.

### 3.2 Silver‐Based NPSs

**Figure 8:**
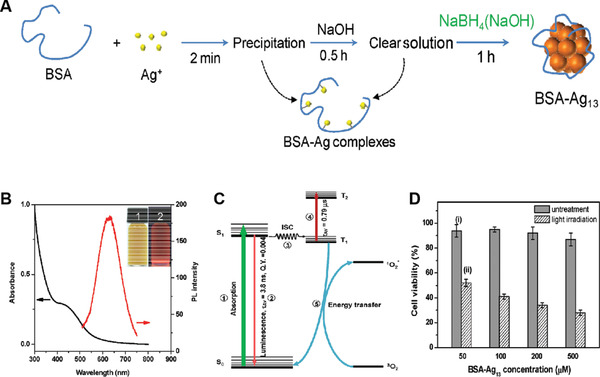
A) Schematic illustration of the new synthesis protocol leading to the formation of BSA‐Ag_13_ NC. B) Photoabsorption (black line) and photoemission (red line, *λ*_ex_ = 480 nm) spectra of as‐synthesized BSA‐Ag_13_ NC. C) Simplified energy diagram of the BSA‐Ag_13_ NC upon photoexcitation in water. S and T or 1 and 3 as superscripts denote singlet and triplet states; 0 and 1 as subscripts denote ground and the first excited states, respectively. D) MTT assay histogram of MCF‐7 cancer cells treated with BSA‐Ag_13_ NC with and without light irradiation. Reproduced with permission.^[^ ^48^ ^]^ Copyright 2017, Wiley‐VCH.

Though the Au NCs have been well studied as efficient NPSs for cancer PDT, the exploration of silver nanoclusters (Ag NCs) for controlling ^1^O_2_ generation is very rare due to the more difficult reduction of Ag^+^ into Ag as well as less stability of Ag NCs to the environment. In 2016, Yu et al. reported an elegant approach for the reliable synthesis of BSA‐templated ultrasmall Ag NCs (BSA‐Ag_13_ NCs), which showed improved stability and extremely high ^1^O_2_ generation capacity for cancer PDT.^[^ ^48^ ^]^ The key strategy utilized in the synthesis of BSA‐Ag_13_ NCs was to dissolve the strong reducing agent NaBH_4_ in an alkaline NaOH solution, which could effectively prevent the decomposition of NaBH_4_ and remove less stable Ag NCs in the final product, thus facilitating to regulate the size and improve the stability of the BSA‐templated Ag NCs (**Figure** **8**A). They demonstrated that the as‐formed BSA‐Ag_13_ NCs exhibited a UV–vis absorption with a shoulder peak at 425 nm and a red emission at 625 nm (Figure 8B), with a fluorescent quantum yield of only 0.4%. Notably, they found that the ^1^O_2_ quantum yield of BSA‐Ag_13_ NCs was much higher (1.26) as compared to its analog BSA‐Au_25_ NCs (0.07) or RB (0.75). The mechanism studies using ultrafast laser spectroscopy like time‐correlated single‐photon counting and transient absorption techniques demonstrated that the majority of BSA‐Ag_13_ NCs could transit to triplet states via intersystem crossing upon photoexcitation (Figure 8C). The subsequent triplet‐triplet transitions could prolong the excited electrons residing at the triplet states, which greatly increased the change to sensitize O_2_ molecules to form ^1^O_2_. Moreover, cellular studies on MCF‐7 breast cancer cells revealed that the BSA‐Au_25_ NCs could easily enter the cells and effectively kill MCF‐7 cells under white light irradiation (Figure 8D). These results suggested that the BSA‐Au_25_ NCs can act as promising NPSs to trigger effective PDT for cancer treatment.

## 4 Metal Oxide‐Based NPSs

Metal oxide NMs have found promising biomedical applications for fluorescent labeling due to their well‐known size dependent physiochemical and optical properties, such as high photostability, large extinction coefficients, high emission quantum yield, and easy surface modifications.^[^ ^49^ ^]^ Previously, metal oxides‐based quantum dots (QDs) have been reported to sensitize the formation of ^1^O_2_ under UV light excitation. However, the inherent cytotoxicity of these QDs due to the presence of heavy metal ions (e.g., cadmium) and low ^1^O_2_ quantum yield restricts their use as a photosensitizer in clinical PDT.^[^ ^50^ ^]^ Alternatively, semiconductor NMs such as titanium dioxide (TiO_2_), zinc oxide (ZnO), copper sulfide (CuS), molybdenum disulfide (MoS_2_), and tungsten‐based nanostructures have also been demonstrated to directly sensitize formation of ^1^O_2_ upon light irradiation. Thus, in the forthcoming section, we will discuss the development of different types of metal oxide NMs as either UV/vis or NIR light triggered NPSs for cancer PDT.

### 4.1 Titanium Oxide

**Figure 9:**
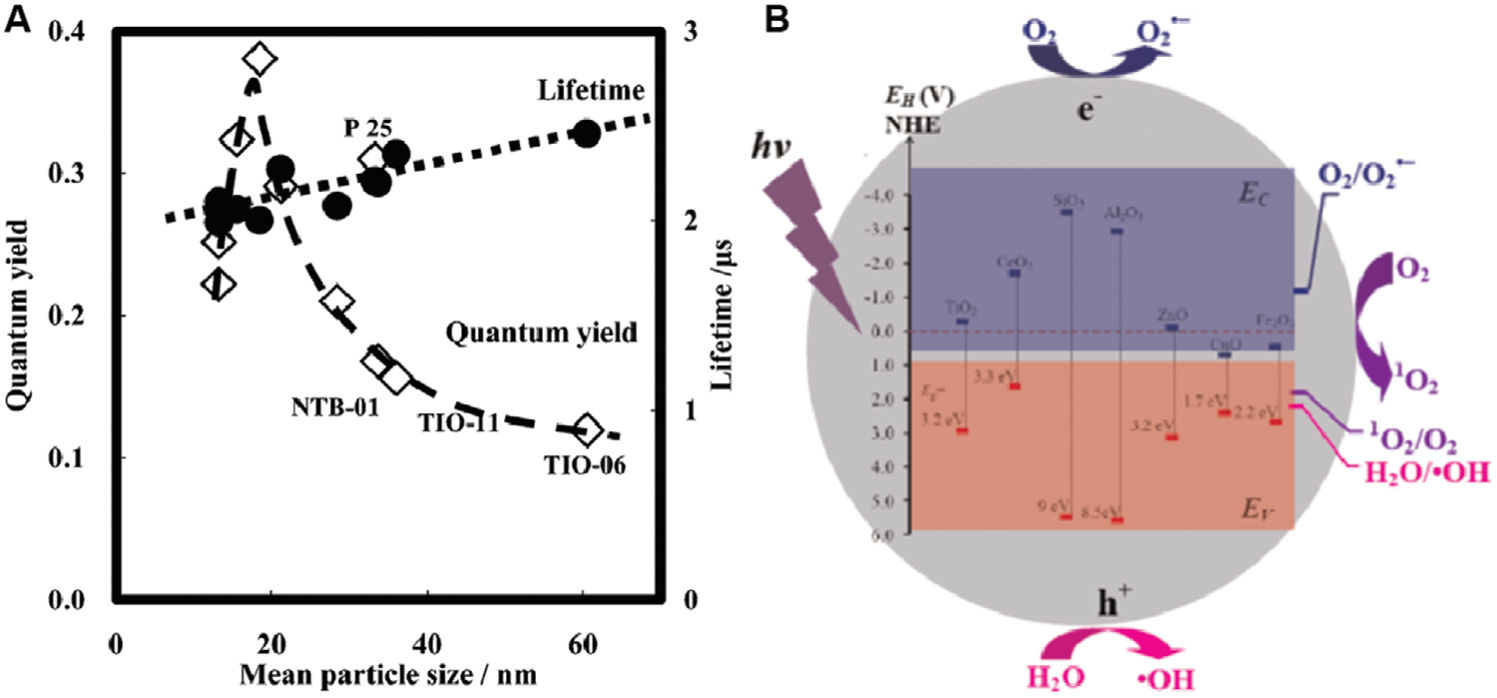
A) Quantum yield and life time of the ^1^O_2_ generated on various TiO_2_ photocatalyst powders in air plotted as a function of the mean size. Reproduced with permission.^[^ ^54^ ^]^ Copyright 2007, American Chemical Society. B) Band edge positions of seven metal oxide in contact with aqueous solution at pH 5.6. The lower edge of Ec (blue) and upper edge of Ev (red) are presented along with the band gap in eV. The blue‐shaded area represents the CB, while the red‐shaded area represents the VB. Reproduced with permission.^[^ ^26^ ^]^ Copyright 2012, American Chemical Society.

In the past few decades, TiO_2_ NPs with a 0D structure have emerged as one of the most extensively studied photocatalysts that have been widely used for organic synthesis, bleaching processes, and the degradation of organic pollutants due to their high ability to generate ROS.^[^ ^51^ ^]^ In addition, TiO_2_ NPs possess excellent photostability and good biocompatibility, which could allow them to be used as NPSs in PDT.^[^ ^52^ ^]^ It is found that irradiation of TiO_2_ NPs under UV light can produce ROS such as O_2_^·−^, OH, H_2_O_2_, and ^1^O_2_, which are highly toxic capable of oxidizing proteins and lipids in cells, finally killing cancer cells. In 2004, Nosaka et al. first reported the direct observation of ^1^O_2_ generation from TiO_2_ NPs under 355 nm pulsed laser irradiation using a gated photon counting method.^[^ ^53^ ^]^ They found that the ^1^O_2_ quantum yield of TiO_2_ NPs was roughly estimated to be ≈0.2 using RB (0.8) as a reference. The plausible mechanism to produce ^1^O_2_ could be due to the photocatalytic oxidation of O_2_^·−^ at the surface of TiO_2_ NPs. It was interesting that the lifetime of ^1^O_2_ generated from TiO_2_ NPs was found to be much higher (5 µs) in ethanol as compared to that in either water (2 µs) or water–ethanol mixture (1:1). They later investigated the formation and behavior of ^1^O_2_ using 10 different commercially available TiO_2_ NPs under different circumstances such as air, H_2_O, D_2_O, and ethanol.^[^ ^54^ ^]^ The lifetimes of ^1^O_2_ from all the 10 TiO_2_ NPs in various environments were found to be very short (2–3 µs), while the ^1^O_2_ quantum yields were in the range from 0.12 to 0.38. They found that the lifetime of ^1^O_2_ in the air was shorter compared to that in H_2_O, but it was longer in ethanol. This difference could be due to the different electronic‐to‐vibrational deactivation processes among them. They also demonstrated that the amount of ^1^O_2_ was the same when the TiO_2_ NPs were present in either H_2_O or D_2_O, due to the similar chemical properties. However, in ethanol suspension, most of the photogenerated holes were consumed during the oxidation of ethanol, resulting in the substantial reduction of the ^1^O_2_ generation_._ Moreover, they found that the ^1^O_2_ quantum yield decreased with increasing size of TiO_2_ NPs when the size is greater than 20 nm (except P25 TiO_2_), which was presumably due to that the amount of O_2_ adsorbed on the surface of TiO_2_ NPs decreased when the particle size increased (**Figure** **9**A)_._ Based on these observations, they proposed a plausible mechanism to illustrate the ^1^O_2_ generation by TiO_2_ NPs upon light irradiation: O_2_ adsorbed on the surface of TiO_2_ NPs is reduced to O_2_^·−^ by an electron in the conduction band (CB), which was followed by oxidation by the hole in the valence band (VB) to form ^1^O_2_. The subsequent study with surface‐modified nanocrystalline TiO_2_ NPs demonstrated a higher ^1^O_2_ quantum yield (0.012) as compared to that of bare TiO_2_ NPs (0.003).^[^ ^55^ ^]^

To get insight into the ROS generation mechanism,^[^ ^26^ ^]^ Li et al. investigated the ROS generation kinetics of seven different metal‐oxide NPs (CeO_2_, Fe_2_O_3_, SiO_2_, Al_2_O_3_, ZnO, CuO, and TiO_2_), and compared them to their bulk counterparts. They demonstrated that TiO_2_ NPs and ZnO NPs produced three types of ROS (O_2_^−^, ·OH, and ^1^O_2_), whereas other metal‐oxide NPs (e.g., CeO_2_, SiO_2_, Al_2_O_3_, and Fe_2_O_3_) produced only one or two types of ROS. CuO NPs did not generate any type of ROS. These results suggested that different metal‐oxide NPs displayed distinct capacity to generate ROS upon light irradiation, with the order of TiO_2_ NPs > ZnO NPs > Al_2_O_3_ NPs > SiO_2_ NPs > Fe_2_O_3_ NPs > CeO_2_ NPs > CuO NPs. Compared to their bulk counterparts, both TiO_2_ and ZnO NPs generated more ROS under UV irradiation, presumably due to that NPs had larger surface areas capable of providing more reaction sites for the absorption of UV light. By comparing the electronic structures of metal‐oxide NPs with the redox potentials of various ROS, they proposed that the band edge structures of metal‐oxide NPs were crucial in the generation of ROS (Figure 9B). As ROS generation is dictated by the photoexcitation‐driven interfacial electron transfer process, only the metal‐oxide NPs with a band gap energy (Eg) smaller than the incident photon energy (i.e., ≈3.4 eV for the 365 nm UV) can be photoexcited to generate excited electrons in CB and holes in the VB, which subsequently react with an aqueous electron acceptor (e.g., molecular oxygen) and donor (e.g., H_2_O and OH^−^), respectively, ultimately producing different types of ROS. Though TiO_2_ NPs have exerted good ROS generation capacity, the requirement of UV light excitation has hampered their applications for in vivo PDT due to the low tissue penetration depth and UV light‐induced damages to normal tissue. Considering that NIR light (650–1300 nm) can penetrate much deeper into tissues as compared to UV light, the incorporation of a light transducer into the TiO_2_ NPs capable of converting NIR to UV light would be beneficial to engineer TiO_2_ NPs as NIR‐driven PSs for in vivo PDT. Hence, upconversion nanoparticles (UCNPs) integration has been adopted to engineer TiO_2_ NPs for the treatment of deep‐seated tumors. Over the past decades, UCNPs made of lanthanide metals have exhibited unique optical properties of absorbing NIR light and emitting UV–vis light, which could serve as an efficient donor capable of transferring UV light to excite the attached TiO_2_ NPs upon NIR light irradiation.^[^ ^56^ ^]^ Accordingly, a number of well‐designed UCNPs with upconversion emission spectra overlapped well with the absorption of the TiO_2_ NPs have been developed, allowing to build UCNP‐TiO_2_ nanocomposites as NIR‐driven ROS producers through an efficient energy transfer process.^[^ ^57^ ^]^ In 2014, Zhang's group reported a uniform core‐shell UCNP‐TiO_2_ nanocomposite consisting of a continuous layer of TiO_2_ coating on individual UCNP core. Under excitation with a 980 nm laser, the UCNP core could efficiently upconvert the 980 nm light to visible and UV light, which can subsequently activate the TiO_2_ layer to produce ROS like O_2_^·−^, ·OH, and H_2_O_2_. They demonstrated that the as‐prepared nanocomposite could enter into oral squamous carcinoma cells (OSCC) and kill more than 50% cells under 980 nm irradiation, indicating a potential NPSs for cancer PDT.^[^ ^58^ ^]^ Based on this work, they later investigated the in vivo therapeutic applications of PEGylated UCNP‐TiO_2_ composite against the OSCC tumor model.^[^ ^59^ ^]^ After intratumoral injection of the PEG‐UCNP‐TiO_2_ into the tumor‐bearing living mice, remarkable inhibition of tumor growth was achieved upon 980 nm light irradiation, which was approximately two to three times lower than the untreated groups. Importantly, no mice were died even after 60 days posttreatment, implying the excellent biocompatibility of the designed nanoconstruct. Meanwhile, Qu's group reported another type of nanocomposite made from a mesoporous TiO_2_ (mTiO_2_) shell coated UCNPs for NIR‐triggered synergistic chemo‐photodynamic cancer therapy.^[^ ^60^ ^]^ In their study, the mTiO_2_ shell in the nanocomposite could be activated by 980 nm light excitation to produce cytotoxic ROS through an efficient energy transfer from UCNP core to mTiO_2_. Moreover, the porous structures of mTiO_2_ shell could also load anticancer drugs (e.g., doxorubicin) for chemotherapy due to the large surface area. They found that the combined PDT and chemotherapy against cultured MDA‐MB‐231 breast cancer cells under 980 nm light excitation was more efficient compared to that of single PDT or chemotherapy.

**Figure 10:**
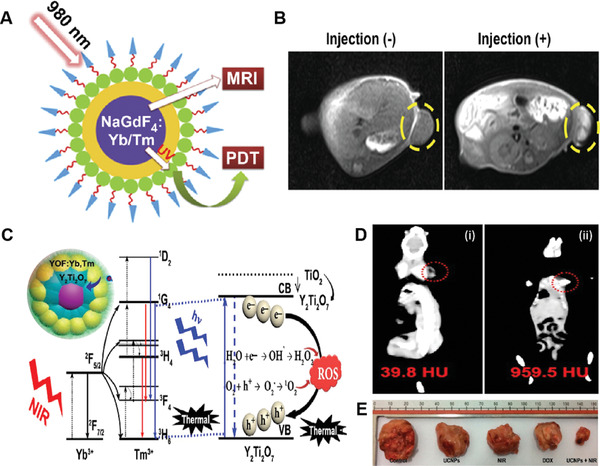
A) Schematic illustration of FA‐Gd‐Si‐Ti NPs for MRI and NIR‐responsive PDT. B) In vivo *T_1_*‐weighted MRI images of MCF‐7 tumor‐bearing nude mice. Reproduced with permission.^[^ ^61^ ^]^ Copyright 2015, Elsevier. C) Schematic diagram of the energy transfer process of TYY UCNPs between Yb/Tm ions and the Y_2_Ti_2_O_7_ photocatalyst. D) CT imaging of tumor‐bearing Balb/nu mice (i) before and (ii) after injection. E) Digital photographs of tumor tissues after different treatments for 14 days. Reproduced with permission.^[^ ^63^ ^]^ Copyright 2015, American Chemical Society.

To realize multimodality imaging‐guided in vivo cancer PDT, Wu's group recently developed folic acid‐decorated NaGdF_4_:Yb/Tm@SiO_2_@TiO_2_ nanocomposites (FA‐Gd‐Si‐Ti NPs) for both magnetic resonance imaging (MRI) and NIR‐triggered PDT of cancer in living mice (**Figure** **10**A).^[^ ^61^ ^]^ In the structure of FA‐Gd‐Si‐Ti NPs, the NaGdF4:Yb/Tm core could serve as both energy donors and *T*_1_‐weighted MR contrast, and the TiO_2_ shell could serve as inorganic NPSs. Moreover, the presence of FA on the surface could selectively bind to the folate receptors overexpressed on some cancer cells, facilitating to enter into cancer cells. They demonstrated that the FA‐Gd‐Si‐Ti NPs had a longitudinal *r*_1_ relaxivity of 4.53 mm ^−1^ s^−1^, enabling to produce bright MR contrast in MCF‐7 tumors (Figure 10B). After intratumoral injection into MCF‐tumor‐bearing mice, the tumor growth was significantly inhibited after irradiation of the MCF‐7 tumors with a 980 nm laser. The tumor growth inhibition ratio was found to be ≈88.6% after 2 weeks, while the average body weights of mice were little changed during the course of treatment. Alternatively, Lin's group reported similar TiO_2_‐coated UCNP core/shell nanocomposites ((NaYF_4_:Yb^3+^, Tm^3+^@NaGdF_4_:Yb^3+^) @TiO_2_) as NIR light‐activated NPSs for imaging‐guided in vivo cancer PDT.^[^ ^62^ ^]^ In their study, the polycrystalline anatase TiO_2_ NPs were successfully coated on the surface of the Yb/Tm‐co‐doped UCNP cores through hydrophilic polymer PVP‐assisted one‐step protocol. They demonstrated that the nanocomposite could be taken up by cancer cells via endocytosis and produce intracellular ROS upon 980 nm NIR laser irradiation, thus causing mitochondrial dysfunction and inducing cell apoptosis. The successful suppression of tumor growth in HeLa tumor‐bearing mice that received intratumoral injection of the nanocomposites and 980 nm laser irradiation was also achieved, suggesting that the strategy of coupling TiO_2_ NPs with UCNPs was effective to sensitize formation of ROS for in vivo cancer PDT under NIR light irradiation. Different from the aforementioned UCNP‐TiO_2_ nanocomplexes that used TiO_2_ as shells, Lin's group latterly reported another folic acid‐decorated core–shell‐nanocomplexes (TiO_2_@Y_2_Ti_2_O_7_@YOF:Yb,Tm) (TYY), in which the TiO_2_ NPs were engineered as the core and the UCNPs of YOF:Yb,Tm with strong blue emissions were employed as the shell.^[^ ^63^ ^]^ Moreover, an intermediate layer of Y_2_Ti_2_O_7_ NPs containing electron acceptors of Y^3+^ was also introduced to improve the photocatalytic activity. Besides ROS generation under 980 nm light excitation, the strong photothermal effect could be simultaneously achieved owing to the nonradiative transition along with the recombination of electron‐hole pairs. Figure 10C illustrates the plausible energy transfer process for the generation of ROS and heat from the TYY UCNPs under NIR light irradiation. Importantly, due to the presence of Yb^3+^ and Y^3+^, the designed TYY UCNPs also showed markedly enhanced CT contrast signal in vivo after injection (959.5 HU) compared to the control group (39.8 HU) (Figure 10D). Under 980 nm laser irradiation, the nanocomplexes showed appreciable combinational PDT and PTT effects by efficiently suppressing tumor growth in living mice (Figure 10E). Thus, TYY UCNPs could offer single NIR‐light triggered CT‐guided combinational in vivo cancer therapy.

Though the coupling of TiO_2_ NPs with UCNPs has shown promising results for in vivo PDT, the requirement of 980 nm NIR light as the irradiation source has brought an inherent drawback for in vivo applications. It is known that human tissues show high absorbance of light at 980 nm, which can shorten the penetration depth and induce overheating effect to cause normal tissue damage. Therefore, engineering of UCNP‐TiO_2_ nanocomposites capable of exciting by NIR light apart from the 980 nm wavelength could be beneficial for safe in vivo PDT, which was successfully demonstrated by Lin's group.^[^ ^64^ ^]^ In their study, a well‐defined core‐shell UCNP‐TiO_2_ nanocomposite (UCNPs@mSiO_2_@TiO_2_) consisting of Nd^3+^‐sensitized UCNPs and TiO_2_ NPs were developed. The doping of Nd^3+^ into the outer layer of the UCNPs core could act as an efficient sensitizer for converting 808 nm NIR light to UV emission. To further improve the upconversion luminescence emission from UCNPs under 808 nm light irradiation, the UCNPs cores were smartly fabricated by introducing a quenching‐shield layer to block the back energy transfer from the inner activator Tm^3+^ to Nd^3+^ in the outer layer. Meanwhile, UCNPs@mSiO_2_@TiO_2_ also possessed dual‐modal (CT/MRI) imaging features, offering high CT value (766.7 HU) after injection than control (23.53 HU) as well as *T*_1_ weighted longitudinal relaxivity (*r*_1_) of 1.588 mm ^−1^s^−1^. In vivo studies showed that the 808 nm light‐triggered PDT efficacy of UCNPs@mSiO_2_@TiO_2_ in living mice was much higher compared to that with 980 nm or UV light. These results suggested that the 808 nm NIR light excitable UCNP‐TiO_2_ nanocomposites could serve as promising NPSs for dual‐modal imaging‐guided safe and efficient cancer PDT.

### 4.2 Tungsten Oxide

**Figure 11:**
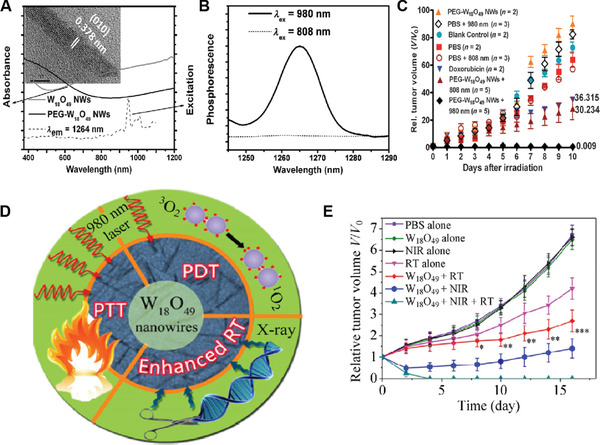
A) UV/vis‐NIR absorption spectrum of W_18_O_49_ NWs before and after PEGylation and excitation spectrum for the phosphorescence of ^1^O_2_ (*λ*_em_ = 1264 nm). B) Phosphorescence signal of ^1^O_2_ emission by PEG‐W_18_O_49_ NWs at 808 and 980 nm light excitation. C) Relative tumor volume in eight different groups as a function of time (days 0–10). Reproduced with permission.^[^ ^65^ ^]^ Copyright 2013, Wiley‐VCH. D) Schematic illustration of the W_18_O_49_ NWs‐based PDT/PTT/RT. E) Tumor response curves under different conditions as a function of time. (*p < 0.05, **p < 0.01, and ***p < 0.001). Reproduced with permission.^[^ ^66^ ^]^ Copyright 2015, Springer.

Tungsten‐based NMs belong to the family of transition metal oxide, are another kind of widely explored biomaterials as their LSPR properties can be easily tuned. Specifically, tungsten oxide‐based 1D nanowires have been of particular interest due to their strong absorption in the NIR regions, high photothermal conversion efficiency, good ^1^O_2_ quantum yield, and large atomic number (*Z* = 73). The first example of using tungsten oxide nanowires as NPS for cancer PDT was demonstrated by Hwang's group in 2013.^[^ ^65^ ^]^ In their study, ultrathin PEGylated W_18_O_49_ nanowires (PEG‐ W_18_O_49_ NWs) with a length of 50 nm, a width of 4 nm, and thickness of 1 nm were prepared, which showed extended absorption in the NIR region up to 1200 nm (**Figure** **11**A). Under 980 nm excitation, the phosphorescence ^1^O_2_ emission signal appeared at 1270 nm, indicating a ^1^O_2_ quantum yield of 0.29. This result suggested that the PEG‐W_18_O_49_ NWs hold a good ability to generate ^1^O_2_ upon 980 nm NIR light irradiation. However, negligible ^1^O_2_ was generated upon irradiation with an 808 nm laser, matching well to the corresponding excitation spectrum (Figure 11B). Cellular studies showed an obvious elevation of ROS levels inside HeLa cells following treatment with PEG‐W_18_O_49_ NWs and 980 nm light irradiation, thus inducing significant cell death. A subsequent in vivo study on B16F0 melanoma tumor‐bearing mice revealed successful destruction of tumors upon PDT with PEG‐W_18_O_49_ NWs and 980 nm light irradiation. On day 10, the average tumor size was only 0.009% of the initial tumor size, which was much smaller compared to that following PTT with 808 nm irradiation (≈30.2%) or chemotherapy with doxorubicin (≈36%) (Figure 11C). Encouraged by this, Qiu et al. latterly reported PVP‐decorated W_18_O_49_ NWs (PVP‐W_18_O_49_ NWs) for combination PDT, PTT, and radiation therapy (RT) of cancers (Figure 11D).^[^ ^66^ ^]^ They demonstrated that the PVP‐W_18_O_49_ NWs could produce remarkable heat and ^1^O_2_ under 980 nm NIR laser irradiation, allowing to elicit PTT and PDT effects to kill tumor cells. Meanwhile, they could also act as radiation dose intensifying agents to enable additional RT due to the presence of heavy element W. The in vivo studies showed that mice administrated with the PVP‐W_18_O_49_ NWs following by irradiation with a 980 nm NIR laser and gamma rays could completely eliminate the subcutaneous 4T1 murine breast tumors (Figure 11E), without tumor recurrence for at least 9 months. These results indicated that the PVP‐W_18_O_49_ NWs with synergistic effects of PDT, PTT, and RT were very promising for cancer therapy.

### 4.3 Bismuth Oxyhalide

**Figure 12:**
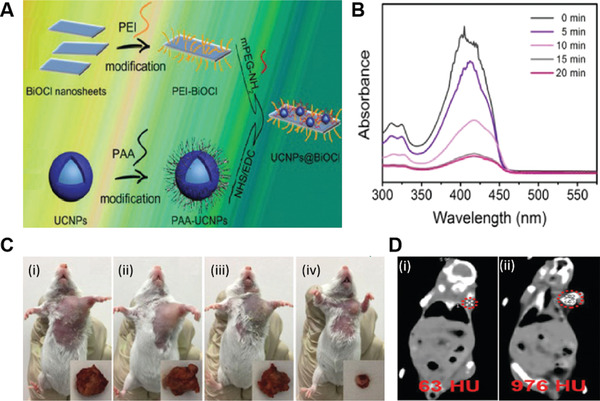
A) Schematic illustration for the formation of UCNPs@BiOCl composite. B) Time course absorbance spectrum of DPBF mixed with UCNPs@BiOCl at a wavelength of 410 nm under a 980 nm laser. C) Representative photographs of tumor‐bearing mice and tumor tissue excised from (i) saline; (ii) pure UCNPs@BiOCl; (iii) 980 nm; and (iv) UCNPs@BiOCl with 980 nm on the 14 day. Reproduced with permission.^[^ ^69^ ^]^ Copyright 2017, Royal Society of Chemistry. D) CT images of tumor‐bearing mice before (i) and after (ii) intratumor injection of BiOBr:Yb,Tm NSs. Reproduced with permission.^[^ ^70^ ^]^ Copyright 2017, Royal Society of Chemistry.

In addition to the previously described titanium and tungsten‐based nanomaterials capable of direct generation of ROS to kill tumor cells upon light excitation, bismuth oxyhalide (BiOCl) is a new emerging metal oxide layered nanomaterial that possesses a tunable bandgap and fascinating physicochemical properties. Because of the unique layered structure, BiOCl showed great potential as a photocatalyst, which can be exploited for light‐mediated therapeutic applications.^[^ ^67^ ^]^ For instance, Xu et al. first investigated the ability of layered BiOCl for cancer PDT.^[^ ^68^ ^]^ In their work, two kinds of BiOCl nanostructures (nanoplates and nanosheets) were prepared by the hydrothermal method and further modified with polyetherimide (PEI) to ensure high aqueous solubility. Under low power UV light irradiation (2.2 mW cm^−2^) for 10 min, BiOCl nanoplates and BiOCl nanosheets showed a dramatic decrease in the MCF cell viability (35% and 70%), respectively. Notably, TiO_2_ did not show any therapeutic effect under this much low power UV irradiation. It has been observed that different electronic band structure and morphology of BiOCl nanoplates and BiOCl nanosheets influenced their cell uptake efficiency, and hence their PDT performance. Second, they possessed different crystal facets (110, BiOCl nanoplates) and (001, BiOCl nanosheets), which strongly affected their in vitro PDT efficacies. Though both the nanostructures showed improved cell killing ability, they did not provide any evidence about the actual ROS specie responsible for inducing cell killing. On the other hand, low penetration depth and potential cytotoxicity associated with UV light remain a challenge in their clinical translation. Therefore, the UCNP@BiOCl nanohybrid was designed by Yang et al. to achieve NIR light‐triggered PDT (**Figure** **12**A).^[^ ^69^ ^]^ Upon 980 nm light irradiation, UCNP@BiOCl showed higher ^1^O_2_ production efficacy compared to UV light, which induced significant tumor cell destruction. This suggested that NIR light could replace UV/vis light for ROS generation (Figure 12B). Moreover, NIR light‐induced in vivo PDT was successfully demonstrated in tumor‐bearing mice (cervical carcinoma cell lines), showing the strongest tumor inhibition effect for mice treated with both UCNP@BiOCl and 980 nm light irradiation in comparison to control groups (Figure 12C). As UCNPs increase the complexity of the system and demand careful consideration of excitation and emission wavelengths, they later developed self‐activated Yb^3+^/Tm^3+^ co‐doped bismuth oxobromide (BiOBr) NSs under 980 nm light irradiation via simple co‐precipitation approach and modified with PEG to endow excellent biocompatibility.^[^ ^70^ ^]^ Due to self‐activation, BiOBr:Yb^3+^/Tm^3+^ NSs revealed high ROS generation capacity, offering exciting in vitro and in vivo therapeutic effects. Besides in vivo cancer therapy, BiOBr:Yb^3+^/Tm^3+^ NSs also possess intrinsic fluorescence as well as due to Yb^3+^/Tm^3+^ co‐doping demonstrated far higher CT value after injection (976 HU) compared to without injection (63 HU) (Figure 12D), respectively, indicating their potential to serve as a dual‐modal imaging agent capable of guiding precise cancer therapy in vivo. As the first demonstration of this kind, these findings will surely pave the way to develop a more self‐activated nanoplatform for non‐invasive deep cancer theranostics.

## 5 Metal Sulfides‐Based NPSs

Apart from TiO_2_ NPs, MoS_2_, and CuS NPs with 0D nanostructures have also been reported to directly generate ROS upon light irradiation,^[^ ^71^, ^72^ ^]^ albeit most of them were widely used as photothermal agents. For example, small‐size fluorescent MoS_2_ QDs with a mean diameter of ≈14.7 nm have been synthesized using a tetrabutylammonium‐assisted sonication approach.^[^ ^73^ ^]^ Upon irradiation with a 630 nm light, the MoS_2_ QDs demonstrated a higher ^1^O_2_ generation ability compared to that of an organic PS, PpIX, as indicated by both the reduction in DPBF absorption and increase in SOSG fluorescence intensity. Though the generation of ^1^O_2_ upon irradiation of MoS_2_ QDs solution was demonstrated, whether the MoS_2_ QDs could serve as PSs to initiate PDT effect either in culture cells or in vivo was still unknown, which will require more biological studies in the future. In another study, Pellegrino and coauthors reported small plasmonic CuS nanocrystals (CuS NCs) that showed both high photothermal and photodynamic properties upon 808 nm NIR light irradiation, which could efficiently suppress B16 subcutaneous tumor growth in living mice.^[^ ^74^ ^]^ Interestingly, they demonstrated that ·OH but not ^1^O_2_ was observed upon irradiation of the CuS NCs, which was mainly due to the heat‐triggered release of Cu(I) ions capable of acting as a source of ROS generation.^[^ ^75^ ^]^ Since the generation of ·OH and heat was independent of O_2_, the CuS NCs might be feasible to treat hypoxic tumors in vivo.

## 6 Carbon‐Based NPSs

In the past few decades, carbon NMs, including 0D fullerenes and carbon dots (C‐dots), 1D carbon nanotubes , and 2D graphene, and graphitic carbon nitride have attracted tremendous attention in biomedical research due to their unique and prominent physicochemical and optical properties.^[^ ^76^ ^]^ For example, carbon NMs have the advantages of chemical inertness, good biocompatibility, high photostability, and tunable fluorescence emission ranging from visible to NIR‐II windows, which make them highly attractive for biosensing and molecular imaging in vivo.^[^ ^77^ ^]^ Recently, well‐designed carbon NMs like fullerenes (C_60_) and GQDs have also been used as NPSs to trigger efficient PDT against tumors due to their high ability to sensitize the formation of ROS. In the following section, we will discuss the recent progress of using carbon NMs as NPSs for cancer PDT.

### 6.1 2D Graphene

Fullerenes are soccer‐ball‐shaped NMs composed of sixty or seventy carbon atoms, which were discovered by Kroto et al in 1985.^[^ ^77^ ^]^ The photophysical properties of fullerenes such as photoabsorption, fluorescence, and phosphorescence have attracted people to explore its potential as NPSs for PDT. However, low solubility under physiological conditions has substantially hindered their applications in biological systems. To overcome this limitation, different functional groups (e.g., OH, COOH, and NH_2_)^[^ ^78^ ^]^ have been introduced into the fullerenes to improve their aqueous solubility. These water‐soluble fullerenes derivatives have shown a high ability to produce ROS like ^1^O_2_, O_2_^−^, and ·OH under photoexcitation,^[^ ^79^ ^]^ which have been successfully applied to elicit PDT activity against viruses,^[^ ^80^ ^]^ bacteria^[^ ^81^ ^]^ and cancer cells.^[^ ^82^ ^]^ For example, Tegos et al. prepared six fullerenes (C_60_) derivatives functionalized with one, two, or three polar diserinol groups or cationic quarternary pyrrolidinium groups, and compared their PDT activities against gram‐positive bacteria, gram‐negative bacteria, and fungi upon white light irradiation.^[^ ^83^ ^]^ They found that the cationic fullerenes derivatives exerted a broad‐spectrum antimicrobial activity, which can rapidly kill more than 99.99% of bacteria and fungi following illumination with white light. The antimicrobial PDT activities were better than that of neutral diserinol group‐modified fullerenes. This was due to the increasing positive charge in the cationic group‐modified fullerenes that could help to bind bacteria and overcome the microbial permeability barriers. These results suggested that cationic fullerenes could be used as effective NPSs for the treatment of localized infections. Besides the antimicrobial PDT activities, they also showed that the cationic group‐functionalized fullerenes could trigger efficient PDT to kill cancer cells via both type‐I and type‐II photochemical processes under illumination.^[^ ^84^ ^]^

**Figure 13:**
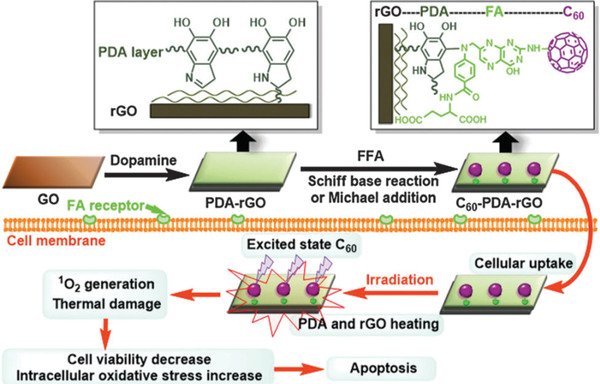
Graphical representation of the fabrication approach of C_60_‐PDA‐rGO nanohybrid, and the possible mechanism of the synergistic effect of combined PTT and PDT. Reproduced with permission.^[^ ^85^ ^]^ Copyright 2014, Royal Society of Chemistry.

In 2014, Hu et al. developed C_60_–PDA–rGO nanohybrids consisting of C_60_ fullerene, polydopamine (PDA)‐coated rGO, and folic acid for targeting PDT/PTT synergistic activities to kill HeLa cells.^[^ ^85^ ^]^ In their study, the graphene oxide was reduced and coated by PDA to form PDA‐rGO, which then subsequently reacted with folic acid‐decorated C_60_ fullerenes (FFA) via a Schiff base reaction or Michael addition. The as‐prepared C_60_‐PDA‐rGO showed a broad absorption extending to NIR regions. Upon irradiation with a Xe lamp equipped with a bandpass filter (400–1100 nm), an obvious production of ^1^O_2_ as indicated by *p*‐nitroso‐*N*,*N*′‐dimethylaniline was observed in the C_60_‐PDA‐rGO aqueous solution. Meanwhile, a remarkable increase in temperature (∆*T* = 17.2 °C) was also achieved. Cellular studies showed that the C_60_‐PDA‐rGO could efficiently enter into HeLa cells via folate receptor‐mediated uptake, and exerted obvious cytotoxicity toward HeLa cells under irradiation by the Xe lamp (**Figure** **13**). It was also found that the phototoxicity induced by C_60_‐PDA‐rGO was higher than that induced by either PDA‐rGO or FFA, suggesting that C_60_‐PDA‐rGO with combined PDT and PTT effects was very promising for synergistic therapy of cancers. Based on this work, they latterly employed host–guest chemistry to develop GO‐FA/Py‐*γ*‐CD/C_60_ nanohybrids, which also showed synergistic PDT/PTT effects to efficiently kill cancer cells under Xe light irradiation.^[^ ^86^ ^]^

In addition to fullerenes, another carbon allotrope, GQDs have also been developed as efficient NPSs for PDT. For example, Markovic et al. reported that GQDs could kill cancer cells by inducing both apoptosis and autophagy (programmed cell death).^[^ ^87^ ^]^ Briefly, GQDs with an average diameter of 56.6 nm and a height of 1.9 nm, respectively, were prepared by an electrochemical approach, showing strong absorption in the UV/vis region and the maximum fluorescence emission at 460 nm. Upon continuous laser irradiation (488 nm, 1 W cm^−2^, 30 min), the prepared GQDs demonstrated a significant amount of ^1^O_2_ as determined by ^1^O_2_ sensitive substrate dihydrorhodamine 123 and EPR spectroscopy. Due to the high ^1^O_2_ generation, GQDs induce oxidative stress which leads to the effective killing of human glioma cells. Notably, increased cell granularity and the downregulation of p62 protein were also observed upon exposure to photo‐irradiated GQDs, suggesting that GQDs could induce both apoptotic and autophagic cell death pathways, which significantly contributed to higher cellular destruction. Importantly, photoexcited GQDs did not show any temperature increase, which clearly rules out the possibility of photothermal killing, suggesting that cellular killing is solely attributed to ^1^O_2_ generation and the activation of dual cell death pathways. Later, the same group reported the antibacterial activity of GQDs against gram‐positive (*S. aureus*) and gram‐negative (*E. coli*) bacteria,^[^ ^88^ ^]^ which also showed a significant reduction in bacterial colonies as observed by the standard plate count method, demonstrating excellent antibacterial activity of GQDs for both bacterial species. Though these findings indicated that GQDs could act as NPSs to initiate efficient anti‐tumor and anti‐bacterial PDT, UV light excitation may damage the cells. To address this issue, Kuo et al. developed TPE GQDs by the ultrasonic shearing reaction, showing an absolute TPE cross‐section of 47903 GM (Goeppert–Mayer).^[^ ^89^ ^]^ They demonstrated that under TPE excitation (808 nm, 2.64 mW)) for only 15 s, GQDs could efficiently produce ^1^O_2_ and O_2_^·−^, which almost completely eliminate both gram +ve and gram −ve bacteria, respectively. Moreover, due to the strong TP luminescence (TPL), GQDs could also serve as a potential contrast agent, enabling imaging‐guided TPE antibacterial PDT. To enhance the ROS generation capacity, the same group further designed one‐photon excited nitrogen‐doped GQDs (N‐GQDs) and TPE amino‐functionalized N‐GQDs with a TPE cross‐section of 54854 GM, which also exhibited intrinsic imaging as well as enhanced TPL character.^[^ ^90^ ^]^ It has been notable that N doping of GQDs as well as amino functionalization of N‐GQDs could readily increase the charge transfer efficiency, which in turn improved the ROS generation capacity, resulting in complete bacterial elimination under one photon (670 nm, 0.1 W cm^−2^) excitation for 3 min and TPE (808 nm, 2.3936 mW) for only 12 s, respectively. Importantly, high antibacterial PDT efficacy could be attributed to high ^1^O_2_ quantum yield, which was found to be 0.60 and 0.53 for N‐GQDs and amino‐N‐GQDs, respectively, compared to 0.41 for unmodified GQDs. Alternatively, another work from Zhang et al. elucidated that the chemical reduction of GOQDs (rGOQDs) could also enhance the ROS generation capacity as a higher yield of ROS including ^1^O_2_, O_2_^−^, and H_2_O_2_ was achieved under white light irradiation compared to GOQDs.^[^ ^21^ ^]^ They suggested that the rGOQDs generated more electron–hole pairs due to the lower bandgap and VB than GOQDs, resulting in improved ROS generation and higher in vitro cancer PDT efficacy. In a subsequent study, Liu et al. provided another strategy to improve ROS yield by coupling GQDs with ZnO. Instead of ^1^O_2_, the resultant hybrid ZnO/GQD showed increased ·OH formation, leading to improved antibacterial effects.^[^ ^22^ ^]^ However, due to the UV light absorption of the hybrid, they could be applied for wastewater treatment but are not suitable for in vivo anticancer therapy. Meanwhile, instead of chemical doping and functionalization, the latter approaches provided an effective way to improve the ROS yield of GOQDs via surface modification and coupling. GQDs were modified with adenine (A‐GQDs) by Wang's group, showing efficient TPE cellular imaging and ^1^O_2_ mediated bacterial killing under white light irradiation.^[^ ^91^ ^]^ Interestingly, A‐GQDs could not get entry into the gram +ve bacteria due to the electrostatic repulsion, and thus A‐GQDs are only effective against gram −ve bacteria. Though exciting antibacterial results have been achieved previously, certain limitations hindered the practical application of GQDs. For example, 1) the requirement of expensive femtosecond laser for TPE imaging‐guided antibacterial PDT; 2) doping of GQDs to enhance ROS generation capacity is a laborious and time consuming process, which demand careful consideration of sophisticated reactions; 3) though GQDs could act as a NPSs to initiate efficient anti‐tumor and anti‐bacterial PDT, their in vivo anticancer PDT applications are remain unverified.

**Figure 14:**
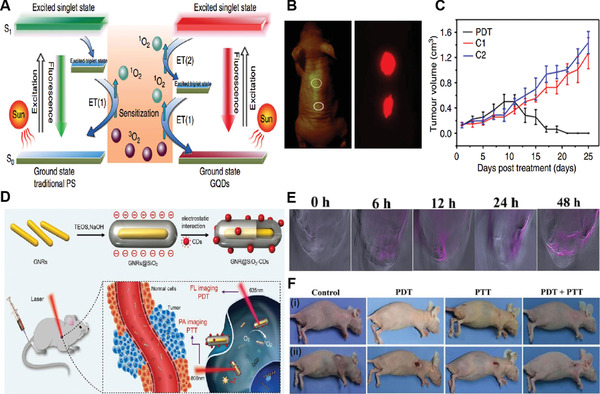
A) Schematic illustration of the ^1^O_2_ generation mechanisms by conventional PDT agents (left) and GQDs (right). B) Bright‐field and red fluorescence image after subcutaneous injection of GQDs in different areas. C) Time dependent tumor growth curves (*n* = 5) after different treatments. *P* < 0.05 for each group. Reproduced with permission.^[^ ^14^ ^]^ Copyright 2014, Nature Publishing Group. D) Schematic illustration of GNR@SiO_2_‐CDs as phototheranostic agent for dual‐modality FL and PA imaging‐guided synergistic PDT/PTT. E) PA images of tumors obtained after i.v. injection of GNR@SiO_2_‐CDs in nude mice at different time points. F) Representative photographs of the four groups after different treatments. Reproduced with permission.^[^ ^92^ ^]^ Copyright 2016, Royal Society of Chemistry.

Bearing this in mind, Ge et al. investigated the in vivo therapeutic applications of GQDs. They developed GQDs by hydrothermal treatment of polythiophene, exhibiting broad absorption ranges from 400–700 nm, deep red emission at 680 nm, and superior photostability than organic dye (PpIX) and CdTe QDs.^[^ ^14^ ^]^ It has been observed that GQDs generate ^1^O_2_ through energy transfer (type‐II) mechanism as evidenced by characteristic ^1^O_2_ ESR signal (1:1:1) and phosphorescent ^1^O_2_ emission signal at 1264 nm. Moreover, the ^1^O_2_ quantum yield was determined by disodium 9,10 anthracendipropionic acid (Na_2_‐ADPA), a chemical trapping agent, revealing that the GQDs possessed an extremely high ^1^O_2_ quantum yield of 1.3, which was possibly due to the proposed multistate sensitization mechanism (**Figure** **14**A). Briefly, according to the absorption and fluorescence spectra, the energy difference between ground state (S0) and excited singlet state (S1) of GQDs was estimated to be 49.3 kcal mol^−1^, and the energy of T1 was calculated to be 22.5 and 26.5^ ^kcal mol^−1^. Whereas, the energy difference between S1 and T1 was estimated to be 22.8 to 26.5^ ^kcal mol^−1^. As the formation of ^1^O_2_ from ^3^O_2_ requires an amount of 22.5 kcal mol^−1^ energy, the ^3^O_2_ received an equal amount of energy on each transition either from S1 to T1 or from T1 to S0 to be converted into ^1^O_2_, resulting in high ^1^O_2_ generation. Due to the high ^1^O_2_ quantum yield, GQDs showed concentration‐dependent toxicity as upon light irradiation using Xe lamp for 10 min, the cell viability decreased from 60% to 20% when the concentration of GQDs increased from 0.036 to 1.8 µm. The in vivo therapeutic efficacy of GQDs against subcutaneous breast cancer xenografts showed that Balb/nu mice with intratumoral injection of GQDs (4 mg kg^−1^) followed by white light irradiation (80 mW cm^−2^) for 10 min could efficiently destroy the tumors after 17 days compared to control groups, which showed significant tumor growth. Interestingly, high in vivo fluorescence intensity with a high signal‐to‐noise ratio was achieved, indicating that GQDs could also serve as an in vivo imaging agent (Figure 14B,C). Consequently, this work suggested that GQDs with excellent biocompatibility and higher ^1^O_2_ quantum yield are a promising candidate for in vivo cancer diagnosis and therapy with enhanced therapeutic efficacy and lesser side effects.

To get multimodal imaging‐guided synergistic PDT/PTT therapy, the same group developed Au NRs@silica‐carbon dots (GNR@SiO2‐CD).^[^ ^92^ ^]^ In the structure, GNRs with a strong longitudinal plasmon band centered at 810 nm could serve as a PA and PTT agent, while CDs act as FL imaging and PDT agent. Meanwhile, the coating of the SiO2 layer onto GNRs avoids the complete fluorescence quenching of CDs. They demonstrated that the GNR@SiO2‐CD could produce remarkable heat and ^1^O_2_ under 808 nm (0.5 W cm^−2^) and 635 nm (0.1 W cm^−2^) laser irradiation, allowing to elicit combined PTT and PDT effects to kill B16‐F0 tumor cells (Figure 14D). In vivo studies on HeLa tumor‐bearing mice following intratumoral injection of GNR@SiO2‐CDs showed remarkably increased FL and PAI signals, and the tumors were completely eliminated under irradiation with 808 nm and 635 nm light irradiation for 10 min, with no apparent side toxicity (Figure 14E,F). Though they demonstrated that synergistic PDT/PTT therapy far better than either PDT or PTT alone, the sequential irradiation by two different lasers to induce combined PDT/PTT effects prolongs the treatment time as well as complicates the treatment process. In addition, it is very hard to precisely align the two laser beams at the same position. Therefore, the development of different strategies or new NPSs capable of inducing combined PDT/PTT effects under single laser irradiation is highly demanded.

**Figure 15:**
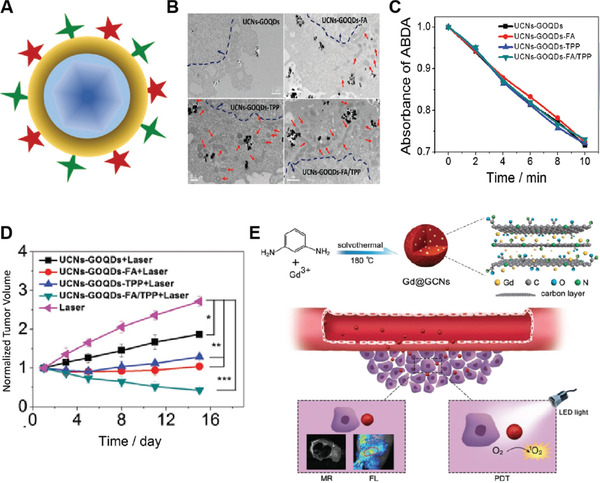
A) Graphical illustration of the design of dual targeted UCNs–GQDs nanohybrid. B) TEM of Hela cells incubated with the nanohybrids. The mitochondria are marked by red arrows and the boundary of nucleus is presented by dashed line. C) ROS generation of UCNs–GOQDs–FA/TPP detected by ABDA. D) The normalized tumor volume under different treatments. Reproduced with permission.^[^ ^23^ ^]^ Copyright 2018, Wiley‐VCH. E) Schematic demonstration of the Gd@GCN as cancer nanotheranostic tools for imaging‐guided phototherapy in solid tumors. Reproduced with permission.^[^ ^94^ ^]^ Copyright 2018, Wiley‐VCH.

Recently, Xing's group covalently attached rhodamine derivative (TRITC) with UCNP‐GQDs hybrid and illustrated mitochondrial‐targeted NIR triggered in vivo PDT.^[^ ^93^ ^]^ Due to the active mitochondrial targeting, UCNP‐GQD/TRITC demonstrated in situ ^1^O_2_ generation within mitochondria under NIR excitation (980 nm), which significantly lower the mitochondrial membrane potential, resulting in the initiation of irreversible tumor apoptotic pathway as verified by the 3.6‐fold enhanced caspase activity for UCNP‐GQD/TRITC compared to 2.5‐fold for non‐targeted UCNP‐GQDs. Moreover, in vivo investigation further revealed effective tumor growth inhibition, suggesting the improved therapeutic performance of UCNP‐GQD/TRITC than UCNP‐GQDs, respectively. Subsequently, synergistic targeting was demonstrated by Liu et al. by attaching folic acid and mitochondrial targeting moiety (caboxylbutyl triphenylphosphonium) with UCNPs‐GQDs nanohybrid to establish synergistic targeting effects (**Figure** **15**A).^[^ ^23^ ^]^ Notably, dual targeting ligands improved the cellular uptake of UCN‐GQDs composite in vitro, showing a 90% increase (3.95 µg per 10^4^ cells) compared to nanohybrid alone (2.08 µg per 10^4^ cells) as further characterized by TEM (Figure 15B). Moreover, under 980 nm laser irradiation (1 W cm^−2^) for 10 min, the designed nanohybrid demonstrates efficient ^1^O_2_ generation efficiency as revealed by the 80% attenuation of ABDA absorption at 400 nm (Figure 15C). Interestingly, the current study employed the strong acid oxidation approach to develop GQDs which possess an ample amount of oxygenated functional groups, enabling efficient O_2_ quenching of triplet states, resulting in increased ^1^O_2_ quantum yield (1.56). Importantly, this is the highest ^1^O_2_ quantum yield ever reported by any organic PS or even inorganic NPS. Due to the highest ^1^O_2_ quantum yield and higher cellular uptake ability, dual‐targeted UCNP‐GQDs nanohybrid exerted significant cell killing and a higher degree of mitochondrial damage compared to either no or single‐targeted UCNP‐GQDs. Meanwhile, in vivo PDT evaluation using female Balb/C nude mice under 980 nm irradiation (0.5 W cm^−2^, 30 min) suggested higher uptake of intraperitoneally injected dual‐targeted nanohybrid (0.21% ID per g) compared to FA (0.15%) or TPP (0.12%) targeted nanohybrid, respectively, resulting in remarkable tumor eradication (Figure 15D). Owing to the dual‐targeting approach, this work will pave the way to improve the accumulation of nanoformulations for better therapeutic performance. In addition, due to the highest ^1^O_2_ quantum yield, the current nanotherapeutic agent could serve as high‐efficiency NPSs for NIR excited deep‐seated tumor PDT. Meanwhile, in another study Chen's group developed ultrasmall (5 nm) gadolinium encapsulated graphene carbon NPs (Gd@GCNs), which could rapidly be cleared from the body through renal clearance, ensuring safe in vivo cancer PDT.^[^ ^94^ ^]^ Under LED irradiation, these Gd@GCNs NPs demonstrated effective ^1^O_2_ generation with a quantum yield of about 0.51, which could effectively destruct cell/tumor both in vitro and in vivo, respectively. In addition, Gd@GCNs showed fluorescence imaging as well as *T*_1_‐weighted MRI with high longitudinal relaxivity (16.0 × 10^−3^ m^−1^ s^−1^), enabling dual‐modal imaging‐guided in vivo PDT (Figure 15E). Collectively, these excellent physicochemical and toxicological characters illustrated that Gd@GCNs is a state‐of‐the‐art nanotheranostic tool with substantial potential for clinical translation.

### 6.2 Graphitic Carbon Nitride

**Figure 16:**
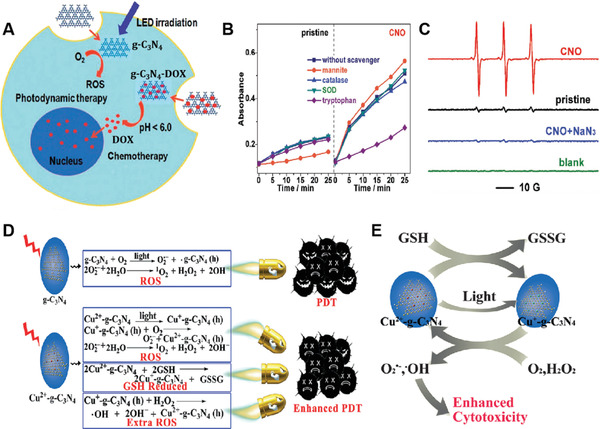
A) Schematic illustration of g‐C_3_N_4_ NSs as potential NPS and pH‐responsive drug nanocarrier for cancer therapy. Reproduced with permission.^[^ ^98^ ^]^ Copyright 2014, Royal Society of Chemistry. B) The absorbance of TMB oxidation with pristine g‐C_3_N_4_ and CNO monitored at 380 nm in the presence of different scavengers. C) ESR spectra of different samples in the presence of TEMP. Reproduced with permission.^[^ ^101^ ^]^ Copyright 2016, Wiley‐VCH. D) Illustration of g‐C_3_N_4_ NSs as NPS for PDT and Cu^2+^‐g‐C_3_N_4_ NSs with enhanced PDT through the synergistic effect of extra ROS generation and GSH depletion. E) Proposed mechanism of GSH reduction and enhanced cytotoxicity. Reproduced with permission.^[^ ^102^ ^]^ Copyright 2016, Wiley‐VCH.

Graphitic carbon nitride nanosheets (g‐C_3_N_4_ NSs) are another newly reported 2D carbon‐based NM, which are mainly composed of carbon and nitrogen atoms.^[^ ^95^ ^]^ Intriguingly, g‐C_3_N_4_ NSs possess high biocompatibility, high photoluminescence (PL) quantum yields, and facile surface modification,^[^ ^96^ ^]^ which could facilitate their biomedical applications. Recently, the use of g‐C_3_N_4_ NSs as NPSs capable of producing ^1^O_2_ for cancer PDT has also been reported.^[^ ^97^ ^]^ For instance, ultrasonic exfoliation of bulk g‐C_3_N_4_ was performed by Lin et al. to develop g‐C_3_N_4_ NSs possessing a lateral dimension of 40 nm and a thickness of 1.1 nm, respectively.^[^ ^98^ ^]^ The resultant g‐C_3_N_4_ NSs showed efficient in vitro ROS generation under LED light irradiation (405 nm, 20 mW cm^−2^, 10 min) as monitored by the bright intracellular green fluorescence from DCFHA, which results in dose and irradiation time‐dependent induction of HeLa cells death. In the meantime, HeLa cells incubated with g‐C_3_N_4_ NSs also demonstrate bright blue color emission under 405 nm excitation wavelength, suggesting the potential of g‐C_3_N_4_ NSs as an imaging agent. Moreover, due to the high surface area to volume ratio of 2D NMs, g‐C_3_N_4_ NSs also offer pH‐dependent drug loading and release of DOX, and thus could act as a pH‐responsive drug nanocarrier (**Figure** **16**A). Meanwhile, both g‐C_3_N_4_ NSs and DOX have been loaded into zeolitic‐imadazolate framework‐8 (ZIF‐8) by Chen et al., which also showed pH‐responsive drug release and ^1^O_2_ generation under visible light irradiation, leading to enhanced therapeutic efficacy.^[^ ^99^ ^]^ Similar to g‐C_3_N_4_ NSs, HA functionalized graphitic hollow C_3_N_4_ nanospheres (GHCNS) also showed imaging‐guided dual‐modal chemo/PDT.^[^ ^100^ ^]^ Overall, these findings illustrated that g‐C_3_N_4_ based nanostructures could serve as a multifunctional nanoagent, offering stimuli‐responsive chemotherapy as well as imaging‐guided PDT. Though g‐C_3_N_4_ NSs have shown promising PDT efficacy in vitro, the lower triplet exciton yield limited their ROSs generation efficiency. It has been noted that triplet exciton yield could be enhanced by incorporating carbonyl groups into the g‐C_3_N_4_ NSs via a simple oxidization approach.^[^ ^101^ ^]^ Interestingly, carbonyl incorporation remarkably reduced the energy gap of singlet to triplet states as well as promoting spin‐orbit coupling, which in turn facilitate higher ^1^O_2_ generation through the type‐II process as well as suppress other ROS species. In contrast, unoxidized pristine g‐C_3_N_4_ only produces ·OH (Figure 16B,C). Conclusively, this work established a new understanding that how the excitonic process influences the formation of ^1^O_2_ in g‐C_3_N_4_, which would be helpful to design NPSs with highly efficient ^1^O_2_ generation. In a subsequent study, Ju et al. suggested that the integration of metal ions (Cu^2+^) into the g‐C_3_N_4_ is also an effective way to enhance the ROS generation capacity.^[^ ^102^ ^]^ As Cu^2+^ integration lowered the energy gap between singlet and triplet states of g‐C_3_N_4_, the resultant Cu^2+^‐g‐C_3_N_4_ NSs could efficiently catalyze the formation of O_2_^·−^ and ·OH from O_2_ and H_2_O_2_, respectively, compared to unmodified g‐C_3_N_4_ NSs. In addition to improved ROS generation, Cu^2+^‐g‐C_3_N_4_ also showed the potential to effectively reduce glutathione (GSH) level by oxidizing GSH into GSSH (Figure 16D,E). As a high level of endogenous GSH significantly consumes the generated ROS, oxidized GSSH could not consume the ROS, which in turn augment the intracellular ROS. Moreover, in the presence of GSH, Cu^2+^‐g‐C_3_N_4_ exhibited enhanced intracellular DCFH fluorescence (74%) compared to g‐C_3_N_4_ NSs (20%), confirming the depletion of GSH level. Thus, Cu^2+^‐g‐C_3_N_4_ illustrated improved in vitro PDT outcomes due to the enhanced ROS generation and efficient depletion of GSH. Though g‐C_3_N_4_ NSs, CNO and Cu^2+^‐g‐C_3_N_4_ hybrid have been demonstrated as potential NPSs for cancer PDT, only in vitro investigation of these NMs has been conducted. Thus, there is still a lack of evidence showing their potential for in vivo cancer treatment. Furthermore, absorption in the UV/vis region significantly limits their in vivo therapeutic applications due to the poor tissue penetration depth of UV/vis light.

**Figure 17:**
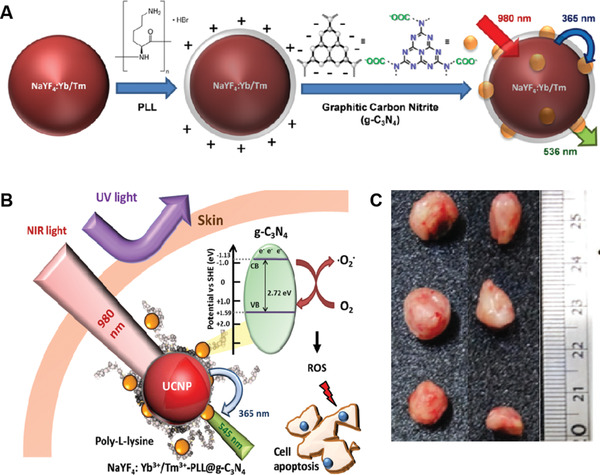
A) Schematic of the synthesis process of UCNP‐PLL@g‐C_3_N_4_ QDs. B) Distinct generation of ROS by UCNP‐PLL@g‐C_3_N_4_ QDs hybrids under NIR illumination and further confirmation of the possibility of adopting such hybrid for PDT. Reproduced with permission.^[^ ^104^ ^]^ Copyright 2016, American Chemical Society. C) Digital images of excised tumors from only NPs group (left) and NP+808 nm light irradiation group (right). Reproduced with permission.^[^ ^105^ ^]^ Copyright 2017, Wiley‐VCH.

To develop NIR excitable g‐C_3_N_4_ based NPSs, Feng et al. integrated UCNPs with g‐C_3_N_4_ NS, which can efficiently convert NIR light (980 nm) and emit UV/vis light that matches well with the absorption of g‐C_3_N_4_ NSs.^[^ ^103^ ^]^ Hence, under 980 nm light excitation, the designed g‐C_3_N_4_/UCNP NCs showed high intracellular ^1^O_2_ generation as verified by the bright green fluorescence of DCFH, which induces significant HeLa cell destruction in vitro. Subsequently, in vivo PDT investigation utilizing the intravenous injection of g‐C_3_N_4_/UCNP NCs (100 mL, 1 mg mL^−1^) into HeLa tumor‐bearing mice was also conducted, showing a remarkable inhibition of tumor growth in mice receiving both g‐C_3_N_4_/UCNP NCs and 980 nm light irradiation (2.5 W cm^−2^). In contrast, control groups showed a negligible growth inhibition effect. Meanwhile, UCL of UCNPs and intrinsic fluorescence character of g‐C_3_N_4_ also offer imaging‐guided in vivo PDT. Thus, these results indicated the suitability of g‐C_3_N_4_/UCNP as a NIR‐triggered theranostic agent. Instead of g‐C_3_N_4_ NSs, UCNPs based g‐C_3_N_4_ QDs (UCNP@g‐C_3_N_4_ QDs and UCNP‐PLL@g‐C_3_N_4_ QDs) hybrid nanoparticles were later designed by Chan et al., which showed O_2_^·−^ generation under NIR excitation (**Figure** **17**A–C), resulting in obvious inhibition of tumor growth both in vitro and in vivo.^[^ ^104^, ^105^ ^]^

## 7 Phosphorus‐Based NPSs

**Figure 18:**
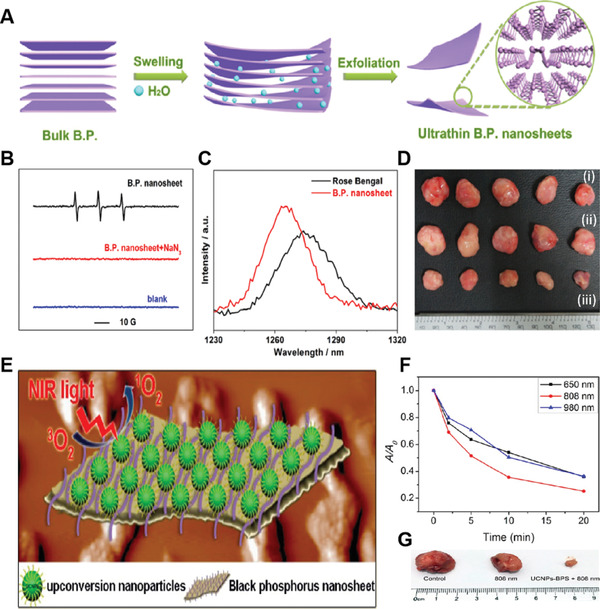
A) Schematic illustration for the water exfoliation of bulk B.P. into ultrathin NSs. B) ESR spectra of B.P. NSs in the presence of TEMP in different conditions. C) ^1^O_2_ emission at ≈1270 nm induced by the commercial RB and B.P. NSs in ethanol under excitation with a 530 nm light. D) Photographs of tumors after treated with various formulations (i) PBS, (ii) BP NSs, and (iii) BP NS + 660 nm light irradiation. Reproduced with permission.^[^ ^107^ ^]^ Copyright 2015, American Chemical Society. E) Diagrammatical representation of NIR based B.P NSs. F) Normalized absorbance of DPBF at a wavelength of 410 nm at different irradiation wavelengths. G) Photographs of tumors on day 14th after different treatments. Reproduced with permission.^[^ ^108^ ^]^ Copyright 2016, American Chemical Society.

Black phosphorus (BP) belongs to the class of attractive 2D inorganic NM that possess sheet‐like morphology with a lateral size of 100 nm to a few µm, while the thickness is within a few atoms (typically <5 µm), tunable band gap, and unusual structural anisotropy. Importantly, structural anisotropic feature significantly contributes to its exceptional optical and electronic properties, which differentiate it from other 2D‐NM, for example, graphene, tungsten diselenide (WSe_2_), MoS_2_, and boron nitride (BN). Though BP has been widely explored in electronic industry,^[^ ^106^ ^]^ unfortunately, little attention has been paid to explore its potential photodynamic therapeutic applications. Hence, studies reported the therapeutic applications of BPs are surprisingly rare. Recently, Wang et al. provided an unprecedented observation that ultrathin BP nanosheets (NSs) could sensitize ^1^O_2_ generation for efficient PDT.^[^ ^107^ ^]^ They performed the exfoliation of bulk BP sample to develop BP NSs with a diameter of about several 100 nm and a height of 2.0 nm, respectively (**Figure** **18**A). The resultant BP NSs demonstrated effective ^1^O_2_ generation efficiency as confirmed by the gradual reduction in DPBF absorbance at 410 nm, an enhanced ESR signal, and the appearance of ^1^O_2_ phosphorescence signal at 1270 nm, respectively (Figure 18B,C). Further, by taking RB as the reference (0.86), the ^1^O_2_ quantum yield of BP NSs was calculated to be 0.91, which was much higher than the most other reported 2D‐NM‐based NPSs. The cellular studies showed that the designed BP NSs exhibited negligible dark toxicity, while a dramatic reduction in cellular viability was observed upon light irradiation at 660 nm (1 W cm^−2^) for 10 min. Subsequently, in vivo PDT evaluation using Balb/c nude mouse after intratumoral injection of BP NSs and light irradiation suggested a significant reduction in tumor growth as compared to control groups (Figure 18D), which is further confirmed by immunohistochemical analysis of tumor tissues.

Though BP NSs showed very high ^1^O_2_ quantum yield and efficient in vivo antitumor effects, the absorption in the UV/vis region does not allow deep penetration, and second, there is a strong concern regarding potential cytotoxicity associated with UV light. To address these issues, Lin's group designed NIR excitable UCNP@BP nanocomposite for deep cancer PDT.^[^ ^108^ ^]^ NaGdF4:Yb,Er@Yb@Nd@Yb UCNPs and BP NSs were first surface modified with polyacrylic acid and *N*‐methyl‐2‐pyrrolidinone, respectively, followed by integrated them by electrostatic interaction to prepare UCNP@BP (Figure 18E). They demonstrated that 808 nm light irradiation was more appropriate to sensitize high 1O2 generation by UCNP@BP than 650 or 980 nm as monitored by a reduction in DPBF absorbance at 410 nm (Figure 18F). Both in vitro and in vivo investigations demonstrated remarkable cell/tumor destruction under 808 nm laser irradiation for 5 min (Figure 18G). Collectively, this study indicated the high ability of UCNP@BP for single NIR laser triggered in vivo deep cancer PDT.

**Figure 19:**
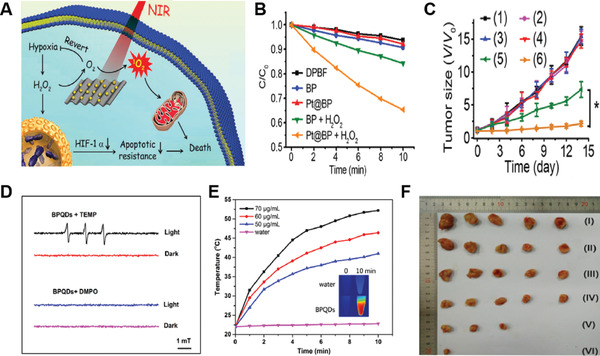
A) Illustration of the antitumor process of Pt@BP nanohybrid. B) Normalized absorbance of DPBF at 410 nm under different conditions. C) Tumor growth curves of different treatments: 1) blank; 2) NIR; 3) BP; 4) Pt@BP; 5) BP+NIR; and 6) Pt@BP+NIR. Reproduced with permission.^[^ ^110^ ^]^ Copyright 2018, Royal Society of Chemistry. D) ESR spectra and E) concentration dependent temperature curves of PEGylated BPQDs under different conditions. F) Photographs of tumors from different groups of mice at 16th day. (I), control; (II), BPQDs; (III), 625 nm light +808 nm laser; (IV), 625 nm light + BPQDs; (V), 808 nm laser + BPQDs; (VI), 625 nm light +808 nm laser + BPQDs. Reproduced with permission.^[^ ^111^ ^]^ Copyright 2017, American Chemical Society.

Though single treatment modality is offering exciting therapeutic results, multimodal therapy could completely eradicate the tumor and thus demonstrate far superior antitumor efficacy than a single treatment approach. Bearing this in mind, Chen et al. developed a multimodal therapeutic platform based on BP NSs, which could induce chemotherapy/PDT/PTT under single laser irradiation.^[^ ^109^ ^]^ They demonstrated that high surface area to volume ratio of BP NSs could allow high drug loading capacity (95% in weight) of electrostatically assembled doxorubicin (DOX, a chemotherapeutic drug). Further, due to the broad absorption from visible to NIR region, BP NSs demonstrated efficient photothermal conversion efficacy as temperature increased from 23 to 45 °C under 808 nm light irradiation (1 W cm^−2^, 3 min). These results suggested that BP NSs could enable the photothermal release of DOX (90% release) to induce chemotherapy as well as could also act as a PTT agent to induce PTT. Besides previously reported BP NSs (PDT agent), the current BP‐DOX nanoplatform could induce synergistic chemotherapy/PDT/PTT under 660 nm and 808 nm, respectively, resulting in significant 4T1 cell/tumor destruction both in vitro and in vivo. Though previous studies efficiently demonstrated the in vivo therapeutic performance of BP NSs, the continuous consumption of tissue oxygen to generate ^1^O_2_ could result in tumor hypoxia, which ultimately limits the PDT efficacy. Incorporation of catalase enzyme into therapeutic formulations is an effective strategy to overcome tumor hypoxia, but enzyme denaturation during both storage and handling is a major concern, which impedes their practical applicability. Alternatively, it is well documented that Pt NPs could act as an artificial catalase enzyme, offering facile synthesis, high stability, excellent catalytic activity, and biocompatibility. Keeping this in mind, Liu's group decorated BP NSs with Pt NPs through an in situ growth approach to overcome tumor hypoxia (**Figure** **19**A).^[^ ^110^ ^]^ Upon the addition of H_2_O_2_ and light irradiation (660 nm), Pt@BP hybrid showed more reduction in DPBF absorption (35%) than either BP NSs alone or Pt@BP hybrid (<10%) without the presence of H_2_O_2_, implying that_._ Pt NPs could efficiently decompose H_2_O_2_ and alleviate tissue oxygen level, resulting in enhanced PDT efficacy (Figure 19B). This was further confirmed by immunohistochemical staining, which revealed the downregulation of hypoxia‐inducible factor (HIF‐1). Due to the effective decomposition of H_2_O_2_, Pt@BP hybrid demonstrated high in vitro and in vivo PDT performance against 4T1 cells/tumor‐bearing mice (Figure 19C), indicating the promising potential of Pt@BP hybrid to treat both normoxic and hypoxic tumor, respectively. As the first demonstration of this kind, this study will promote the development of more therapeutic formulation exclusively based on nanomaterials to treat hypoxic tumors. Instead of BP NSs, Li et al. reported the design of BP quantum dots (BP QDs) as theranostic probes for bioimaging and synergistic PDT/PTT of cancers.^[^ ^111^ ^]^ They used a liquid exfoliation approach to prepare BP QDs with a mean diameter of 2.5 nm, which was further modified with hydrophilic PEG5000 polymers to improve their water solubility and biocompatibility. The synthesized BP QDs exhibited a broad absorption band across the UV and NIR regions, and good photoluminescence property (excitation = 500 nm, emission = 577 nm), indicating the potential for bioimaging. The ^1^O_2_ generation was verified by DPBF and ESR spectroscopy and the ^1^O_2_ quantum yield was found to be ≈0.74 as monitored by PL spectra (Figure 19D). In addition to ^1^O_2_ generation, BP QDs also showed significant temperature increase, which indicated that they could also be used as a PTT agent (Figure 19E). The combined in vitro PDT/PTT cellular studies showed that BP QDs could enter HepG2 liver tumor cells, causing more than 80% cell death upon irradiation with 625 nm (80 mW cm^−2^, 10 min) and 808 nm (2 W cm^−2^, 2 min) laser for combined PDT/PTT, demonstrating superior synergistic anticancer efficacies over single treatment modality. Moreover, the combinational PDT/PTT modalities could also allow BP QDs to efficiently eradicate tumors in 4T1 tumor‐bearing mice (Figure 19F). Importantly, due to their small size, BP QDs could easily and rapidly remove from the body without exerting any potential cytotoxicity, thus offering safe in vivo cancer therapy.

For instance, Guo et al. verified the renal clearance of BP QDs by monitoring the P content in urine.^[^ ^112^ ^]^ They demonstrated that more than 65% of BP QDs were found in the urine within 8 h of intravenous injection, confirming their effective clearance through the excretory system. Further, TEM analysis of BP QDs collected from urine sample show the same morphology and hydrodynamic size, indicating their intact nature, which could allow safe long‐term mediated cancer PDT. Meanwhile, under 670 nm light irradiation, BP QDs effectively generate ^1^O_2,_ which could result in anti‐cancer PDT effects both in vitro and in vivo. In addition to combined PDT/PTT, Zhang et al. developed MRI guided synergistic PDT/PTT platform by assembling Fe_3_O_4_‐carbon dots (Fe_3_O_4_‐CD) with BP QDs and revealed that the resultant Fe_3_O_4_‐CD@BP possess high transverse relaxivity (*r*_2_) of 50.867 × 10^−3^ m^−1^ s^−1^ with a concentration‐dependent darkening effect, enabling enhanced *T*_2_ MRI in vivo.^[^ ^113^ ^]^ Taken together, such observations evidently demonstrated that BP QDs could serve as an effective theranostic agent capable of diagnosis as well as initiate synergistic PDT/PTT against cancers under NIR light irradiation. Though combined PDT/PTT activity of BP QDs has been successfully demonstrated in vivo by different groups, the requirement of two different laser is a major concern in their practical clinical application. Therefore, the development of BP QDs which could trigger combine PDT/PTT under single NIR laser is highly demanded.

## 8 Hybrid‐Based NPSs

Though metal oxides especially TiO_2_ have shown remarkable biomedical applications both in vitro and in vivo as we have discussed in detail in the previous section. However, absorption in the UV region due to the high band gap is a major hurdle in the practical clinical applications of TiO_2_ based NPSs. To address this issue, UCNPs have been integrated with TiO_2_ based NPSs as we discuss several recent examples of this approach in the previous section. However, the integration of UCNPs increases the complexity of the system and requires careful consideration of excitation and emission wavelengths. Alternatively, plasmonic metal nanocrystals have high absorption/scattering cross‐sections and therefore, can enhance and extend the light absorption of TiO_2_ NSs through scattering, absorption enhancement, and hot electron injection.^[^ ^114^ ^]^ Encouraged by this, plasmonic metal NSs have been successfully incorporated into TiO_2_ NSs to harvest visible or NIR light for the generation of ^1^O_2_ and other ROS species_._ Therefore, hybrid NSs are another class of attractive NMs that have been exploited as NPSs to overcome the shortcomings of TiO_2_ based NPSs.

**Figure 20:**
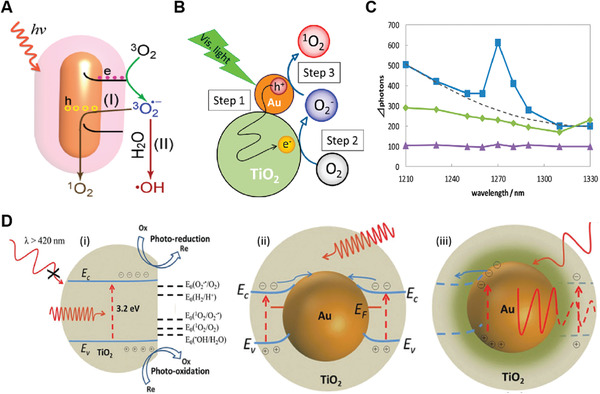
A) Proposed mechanism of ^1^O_2_ by Au NRs core/TiO_2_ shell nanocomposite under 809 nm light illumination. Reproduced with permission.^[^ ^24^ ^]^ Copyright 2014, Royal Society of Chemistry. B) Plausible mechanism for the generation of O_2_^·−^ and ^1^O_2_ on AuNP/TiO_2_ excited with visible light. C) Phosphorescent ^1^O_2_ emission spectra for AuNP/TiO_2_ (blue ■), bare TiO_2_ (green ◆), and AuNP (purple ▲). Reproduced with permission.^[^ ^25^ ^]^ Copyright 2014, American Chemical Society. D) Mechanistic illustration of the enhanced generation of charge carriers of ROS in Au@TiO_2_ (i). However, the enhancement mechanism is different under full light (ii) and visible light (ii). *E*_c_ and *E*_v_ represent the CB and VB of TiO_2_, respectively. *E*_f_ represents the Fermi level of Au. Reproduced with permission.^[^ ^115^ ^]^ Copyright 2018, Royal Society of Chemistry.

In 2014, Fang et al. reported Au/TiO_2_ core–shell NSs for the plasmon‐enhanced generation of ROSs.^[^ ^24^ ^]^ In this work, the TiO_2_ shell was coated onto the pregrown Au NRs to develop Au/TiO_2_ core–shell hybrid NSs. The resultant hybrid NSs have an average diameter of 32 nm and a length of 91 nm, respectively. The ^1^O_2_ generation efficiency of the Au/TiO_2_ core–shell NS was determined by ADBA, showing a 43% reduction in ADBA absorbance after 2 h NIR laser illumination (809 nm, 8.5 W cm^−2^) as compared to 10% for the uncoated Au NRs and hollow TiO_2_, respectively. This suggested that a significant amount of ^1^O_2_ was generated by Au/TiO_2_ core–shell NS in a time‐dependent manner. In addition to ^1^O_2_, ·OH was also detected using terephthalic acid (TA), which can react with ·OH to generate a fluorescent product. However, the fluorescence intensity of TA was five times stronger than uncoated Au NRs and hollow TiO_2_ NSs after 2 h of illumination, indicating that the LSPR phenomenon of plasmonic metal (Au NRs) plays a key role in enhanced ROS generation from core/shell structure. In addition, the recombination efficiency of photogenerated charge carriers (electron–hole pairs) is significantly lower in the core/shell structure than individual NSs, leading to enhanced ROS generation. They proposed the ROS generation mechanism from the hybrid NSs: under plasmon resonant excitation of Au NRs, electrons having energies higher than that of the Schottky barrier could be directly transferred into the CB of TiO_2_ and reduced the adsorbed O_2_ molecules on the surface of TiO_2_ to generate O_2_^·−^. The resultant O_2_^·−^ interact with the remaining hole in the Au NRs or surrounding water to generate ^1^O_2_ or ·OH (**Figure** **20**A). Encouraged by this finding, Saito et al. reported another example of Au NPs/TiO_2_ hybrid NSs and studied the influence of Au loading onto TiO_2_ and the effect of TiO_2_ particle size on ROS generation. They used 13 commercially available TiO_2_ powders to develop Au NPs/TiO_2_ hybrid NSs. They revealed that under visible light irradiation, Au NPs/TiO_2_ hybrid NSs could also produce both O_2_^−^ and ^1^O_2_^[^ ^25^ ^]^ with a quantum yield of 8 × 10^−6^ and 2 × 10^−6^, respectively. Additionally, the phosphorescent ^1^O_2_ emission signal at 1260 nm further verified ^1^O_2_ generation from the Au NPs/TiO_2_ hybrid. Moreover, the mechanism of O_2_^−^ and ^1^O_2_ generation from Au NPs/TiO_2_ was similar as proposed by Feng et al. (Figure 20B,C). Importantly, Au NPs/TiO_2_ hybrid containing larger TiO_2_ particle size generates more O_2_^·−^ and ^1^O_2_ due to the slow recombination of electron–hole pairs in larger Au NPs/TiO_2_ than smaller size TiO_2_ as confirmed by laser spectroscopy. Alternatively, a higher amount of Au loading on TiO_2_ seriously impeded the adsorption of oxygen molecules on the surface of TiO_2,_ leading to low ^1^O_2_ generation. In another subsequent study, instead of electron transfer from Au NPs to TiO_2_, Meng's group suggested that Au NPs/TiO_2_ hybrid behaves differently under different light excitation (sunlight and visible light).^[^ ^115^ ^]^ They demonstrated that under sunlight irradiation, an electron could also be injected from TiO_2_ to Au NPs, while under visible light irradiation (>420 nm), SPR induced near field enhancement effect as well as hot electron injection from Au NPs to TiO_2_ are dominant as described in previous studies (Figure 20D). Both the enhancement mechanisms lead to a dramatic increase in the generation of ROS species (·OH and ^1^O_2_) and charge carriers (electron and hole) as monitored by ESR spectroscopy. Though metal/semiconductor hybrid NSs demonstrated an efficient ROS generation especially ^1^O_2_ particularly in the context of PDT, their suitability for in vitro and in vivo PDT are still unexplored.

**Figure 21:**
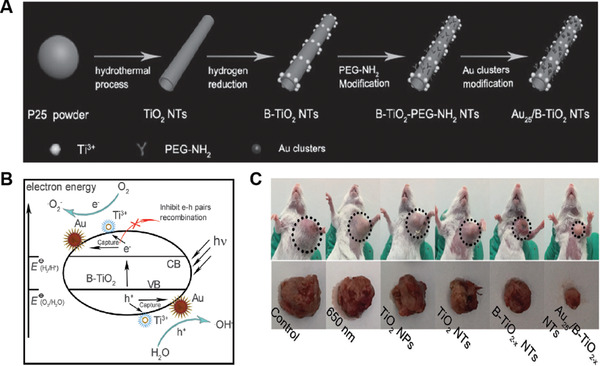
A) Schematic process for the synthesis of Au_25_/B‐TiO_2−_ *_x_* NTs. B) The photocatalytic water splitting mechanism on Au_25_/B‐TiO_2−_ *_x_* NTs. C) Representative photographs of tumor‐bearing mice and tumor from those treated with different conditions upon 650 nm laser irradiation on the 14th day. Reproduced with permission.^[^ ^27^ ^]^ Copyright 2017, Wiley‐VCH.

Recently, in vivo PDT evaluation of Au nanoclusters (Au_25_ NC) stabilized black TiO_2_ nanotubes (B‐TiO_2_ NTs) were first reported by Yang et al.^[^ ^27^ ^]^ Using TiO_2_ NPs as the substrate, TiO_2_ NTs with a thickness of 2 nm and a width of 15 nm, respectively, were prepared by the hydrothermal approach and then undergo high pressure gaseous (H_2_) reduction to develop B‐TiO_2_ NTs. They suggested that the reduction of anatase TiO_2_ could extend the absorption of TiO_2_ NTs from UV to the visible and even NIR region, allowing deeper tissue penetration depth. On the other hand, the reduction process increased the density of Ti^3+^ on the surface of B‐TiO_2_ NTs, enabling effective suppression of electron–hole pairs recombination. Moreover, the reduced B‐TiO_2_ NTs were further modified with Au_25_ NC to develop hybrid NSs (**Figure** **21**A). Interestingly, the deposition of Au_25_ NC further hampered the recombination of charge carriers by changing the electrical distribution in the hybrid, demonstrating enhanced ROS generation efficacy. Instead of ^1^O_2_ generation, Au_25_/B‐TiO_2_ NTs generate efficient O_2_^·−^ and ·OH (Figure 21B), which could induce more than 80% cell death relative to the B‐TiO_2_ NTs or TiO_2_ NTs under 650 nm light illumination (0.5 W cm^−2^, 10 min). This suggested that the Au_25_ NC and the increased density of Ti^3+^ could trigger an improved PDT effect to kill tumor cells. The subsequent in vivo studies in murine cervical cancer mice were performed by intravenously injected Au_25_/B‐TiO_2_ NTs following irradiation with a 650 nm laser, showing significant destruction of tumors, which was more effective compared to the B‐TiO_2_ NTs or TiO_2_ NTs alone (Figure 21C). As the Au_25_/B‐TiO_2_ NTs possessed excellent in vivo PDT performance at NIR regions, the hybrid NSs reported in this work could serve as outstanding NPSs for effective cancer PDT.

**Figure 22:**
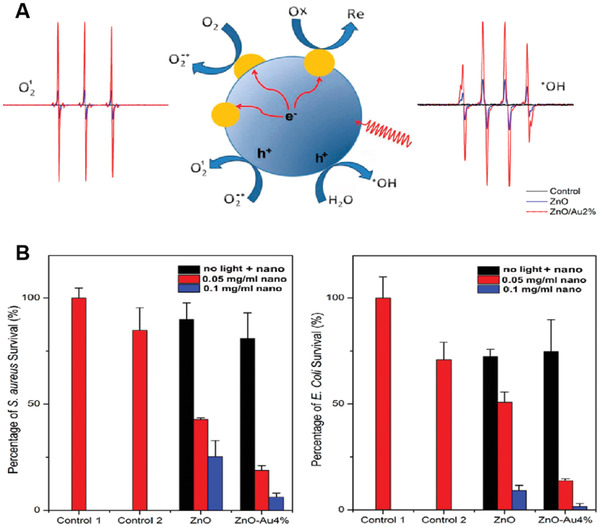
A) Expected reaction mechanism for enhancement effect on generation of ROSs and photocatalytic activity. Corresponding enhanced ESR spectra of ^1^O_2_ (left) and ·OH (right), respectively. B) Ability of ZnO NPs and ZnO/Au hybrid nanostructures in killing *S. aureus* and *E. coli* under simulated sunlight for 10 min. Reproduced with permission.^[^ ^116^ ^]^ Copyright 2014, American Chemical Society.

Instead of using TiO_2_, He et al. developed zinc oxide (ZnO)/Au hybrid NSs (ZnO/Au) and investigated their photocatalytic and antibacterial activity.^[^ ^116^ ^]^ They employed the photoreduction approach, in which AuCl_4_^−^ were reduced by photogenerated electrons from the ZnO, resulting in ZnO/Au hybrid. This method offers two advantages: 1) smaller size Au NPs (<3 nm) on the surface of ZnO, and 2) this approach is eco‐friendly and cost‐efficient as it does not require any additional reactants. They further revealed the injection of an electron from semiconductor (ZnO) to metal (Au) under photoexcitation (**Figure** **22**A), leading to suppress electron–hole pairs recombination, resulting in enhanced ROS generation as confirmed by four times higher photocatalytic degradation of methylene blue (MB) and salicylic acid (SA) by hybrid NSs than ZnO NPs alone. To get a clear insight, ESR spectroscopy with spin trapping and spin labeling agents was employed to investigate the actual ROS species produced by ZnO/Au hybrid and photogenerated charge carriers (electron, hole). They demonstrated that three ROS species including O_2_^·−^, ·OH, and ^1^O_2_ were produced by hybrid NSs upon 5 min irradiation as verified by a characteristic four‐line spectrum 1:2:2:1 and a three‐line spectrum 1:1:1, respectively. It is notable that the increased amount of Au loading onto ZnO significantly enhanced the photocatalytic performance of ZnO. Due to the enhanced ROS generation capacity, the antibacterial activity of ZnO/Au hybrid was also evaluated against gram‐positive *S. aureus* and gram‐negative *E. coli*, showing that hybrid NSs were about three times more effective in killing bacterial cells as compared to pure ZnO NPs (Figure 22B). Due to high power white light excitation, the present hybrid NSs is not suitable for in vivo cancer PDT, but holds a promising potential for use in water purification and antibacterial products. Though in vitro and in vivo PDT applications of hybrid NSs are not widely explored, the current observations and detailed mechanistic investigations will truly pave the way to explore more hybrid NSs (e.g., Au/MoS_2_) for ROS generation as well as the development of advanced strategies to tune the absorption of hybrid NSs in NIR region for in vivo cancer PDT.

**Figure 23:**
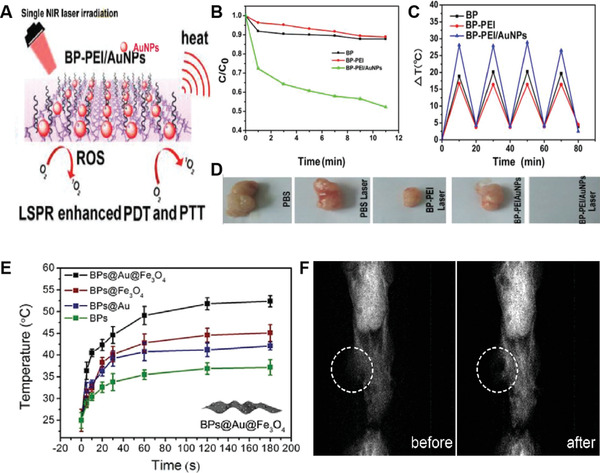
A) Schematic view of the biofunction of BP‐PEI/AuNPs. B) Normalized absorbance of ABDA at 380 nm during photodecomposition by ROS generation upon 670 nm laser irradiation under different materials. C) Temperature elevation curves of different solutions over four rounds of on/off cycling under 670 nm laser at a power density of 1 W cm^−2^. D) Tumor photographs after indicated treatments at 14th day. Reproduced with permission.^[^ ^117^ ^]^ Copyright 2018, Wiley‐VCH. E) The corresponding temperature profiles of the HeLa cells incubated with indicated materials as a function of 650 nm laser irradiation time (0.5 W cm^−2^). F) In vivo MR images of a mouse before and after injection of BPs@Au@Fe_3_O_4_. Reproduced with permission.^[^ ^118^ ^]^ Copyright 2017, Wiley‐VCH.

In another study, Zhang et al. integrated plasmonic Au NPs with PEI functionalized 2D BP NSs through electrostatic interaction to enhance the ^1^O_2_ generation capacity and photothermal performance of BP NSs (**Figure** **23**A).^[^ ^117^ ^]^ As metal NPs possess an exciting LSPR phenomenon which could enhance the light absorption, as well as ^1^O_2_ generation ability of nearby PS via local field enhancement effect, BP‐PEI/Au hybrid presented 3.9‐fold enhanced ^1^O_2_ generation and 1.7‐fold high PTT efficiency of BP NSs compared to BP‐PEI alone under 670 nm laser irradiation (Figure 23B,C). Additionally, in vitro and in vivo studies using HepG2 cell/tumor mice further verified the enhanced dual‐modal PDT/PTT therapy as complete tumor eradication was achieved without any side effects (Figure 23D). This study highlighted the potential of plasmonic materials to incorporate with either 2D NPSs or other NPSs to enhance therapeutic performance. Encouraged by this, Lin's group combined Fe_3_O_4_ NPs with BPs@Au for MRI guided synergistic PDT/PTT.^[^ ^118^ ^]^ The BPs@Au@Fe_3_O_4_ composite was developed via simple electrostatic attraction among BP NSs, Au@glutathione (Au@GSH), and Fe_3_O_4_@polyetherimide (Fe_3_O_4_@PEI) NPs, showing higher ROS production as well as higher photothermal conversion efficiency under 650 nm light irradiation (0.5 W cm^−2^) compared to BP NSs alone, BPs@Au, and BPs@Fe_3_O_4_. This suggested that both Au and Fe_3_O_4_ NPs could participate synergistically to enhance the photothermal performance, resulting in high PTT efficacy of BPs@Au@Fe_3_O_4_ composite (Figure 23E). Owing to the tumor targeting and MRI‐guiding ability of Fe_3_O_4_ NPs, a high transverse relaxivity (*r*_2_) of 54.5 × 10^−3^ m^−1^ s^−1^ was achieved with a concentration‐dependent darkening effect as monitored by *T*_2_‐weighted MRI studies (Figure 23F). Further, a significant visible signal attenuation was observed in vivo, indicating the potential of MR contrast enhancement effect of BPs@Au@Fe_3_O_4._ Moreover, intravenous injection of BPs@Au@Fe_3_O_4_ exhibited superior magnetic remote control (synergistic PDT/PTT) in vivo therapeutic effects against U14 tumor‐bearing mice and excellent biocompatibility as revealed by H&E staining, serum biochemistry assay, and biodistribution studies.

**Figure 24:**
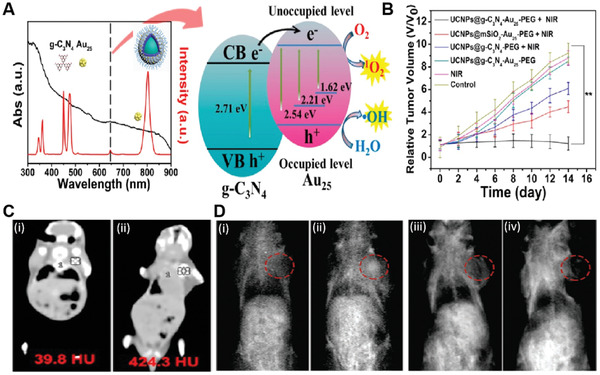
A) UV/vis absorption (black) and emission (red) spectrum of UCNPs@g‐C_3_N_4_‐Au_25_‐PEG upon 980 nm laser excitation and schematic illustration for the ROS generation mechanism of UCNPs@g‐C_3_N_4_‐Au_25_‐PEG irradiated at 980 nm NIR light. B) The relative tumor volume under different conditions as mentioned. C) CT imaging and 3D renderings of CT images of a tumor‐bearing Balb/c mouse: pre‐ (i) and post‐injection (ii). Reproduced with permission.^[^ ^119^ ^]^ Copyright 2017, American Chemical Society. D) In vivo *T_2_/T_1_* weighted MRI images of a tumor bearing Balb/nu mice, pre‐injection (i),(iii) and post‐injection (ii),(iv), respectively, of Fe_3_O_4_@UCNPs@g‐C_3_N_4_. Reproduced with permission.^[^ ^120^ ^]^ Copyright 2017, Wiley‐VCH.

Apart from single ^1^O_2_ NPSs, Feng et al. first time designed dual‐inorganic NPSs (g‐C_3_N_4,_ Au_25_ NCs) based strategy for multimodal imaging and cancer PDT.^[^ ^119^ ^]^ The UCNPs@g‐C_3_N_4_‐Au_25_‐PEG was fabricated via surface coating of UCNPs with a mesoporous g‐C_3_N_4_ layer, and followed by attaching Au_25_ NCs via electrostatic interactions. Under 980 nm excitation, UCNPs emit both UV/vis and NIR light, which can excite the attached g‐C_3_N_4_ and Au_25_‐PEG, respectively, to produce ROS (**Figure** **24**A). Importantly, the prepared UCNPs@g‐C_3_N_4_‐Au_25_‐PEG hybrid effectively promotes the electron–hole pair separation, and thus exhibited high ^1^O_2_ generation ability with a quantum yield of 0.74, allowing to induce remarkable cellular/tumor destruction both in vitro and in vivo, respectively (Figure 24B). It has been noted that UCNPs@g‐C_3_N_4_‐Au_25_‐PEG do not produce significant heat at 980 nm light irradiation both in vitro and in vivo, suggesting that the cell death is solely attributed to high ^1^O_2_ generation. In addition to UCL imaging, the currently developed UCNPs also exhibited a longitudinal (*r*_1_) relaxivity of 0.9195 mm ^−1^ s^−1^ with a concentration‐dependent brightening effect as well as a far superior CT value of 424.3 HU (Hounsfield unit) after injection compared to without injection (39.8 HU) (Figure 24C), respectively, making them a suitable candidate to serve as a *T*_1_ weighted MRI/CT contrast agent. Moreover, the ability to perform simultaneous tri‐modal imaging with UCNPs@g‐C_3_N_4_‐Au_25_‐PEG could offer 3D whole‐body and high‐resolution imaging to guide precise cancer therapy in vivo. Inspired by this, they also reported enhanced *T*_1_/*T*_2_ weighted MRI from Fe_3_O_4_@UCNPs@g‐C_3_N_4_ system with a calculated longitudinal (*r*_1_) and transverse (*r*_2_) relaxivity of 2.545 and 23.080 mm ^−1^ s^−1^, respectively (Figure 24D).^[^ ^120^ ^]^ Due to an improved magnetic guided accumulation and type‐I ROS species generation, Fe_3_O_4_@UCNPs@g‐C_3_N_4_ completely inhibited the tumor growth without any perceived adverse effects. In conclusion, these findings depicted the theranostic applications of g‐C_3_N_4_‐based nanoconstructs, which will truly shine some light on the development of high‐efficiency NIR excitable g‐C_3_N_4_‐based 2D‐NMs as NPSs for multimodal imaging‐guided cancer therapy.

**Figure 25:**
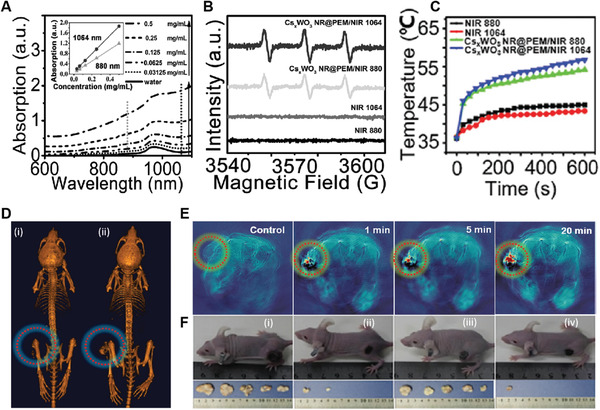
A) Absorption spectra of Cs*x*WO_3_ NR@PEM aqueous suspension with different concentrations and corresponding linear fit between concentration and absorption (inset: the top curve represents absorption intensity at 1064 nm and the bottom curve represents absorption intensity at 880 nm). B) ESR spectra of Cs*x*WO_3_ NR@PEM with TEMP probes under 10 min irradiation. C) Temperature variation of tumors on different groups during laser irradiation. D) CT images of mice before (i) and after (ii) intratumoral injection of Cs*x*WO_3_ NR@PEM (4 mg mL^−1^, 50 µL). E) In vivo PAT images of HeLa‐tumor‐bearing mice before and after intratumoral injection (2 mg mL^−1^, 50 µL) for different times. F) Representative photos of HeLa‐tumor‐bearing mice and tumors after 14 d treatment with Cs*x*WO_3_ NR@PEM under 808 nm (i) 5 min, (ii) 10 min and 1064 nm (iii) 5 min, and (iv) 10 min, respectively. Reproduced with permission.^[^ ^121^ ^]^ Copyright 2016, Wiley‐VCH.

Instead to gold‐based hybrids, Guo et al. reported tungsten‐based metal semiconductor hybrid consists of polyelectrolyte multilayers (PEM) coated cesium tungstate nanorods (Cs*x*WO_3_ NRs@PEM) as a multifunctional nanotheranostic agent for bimodal (CT/PAI) imaging‐guided PDT/PTT of tumors.^[^ ^121^ ^]^ In their study, Cs*x*WO_3_ NRs@PEM with a mean diameter of 13 nm and a length of 70 nm, respectively, were prepared, showing strong absorption in NIR regions that cover both biological windows I and II (**Figure** **25**A). They demonstrated that the strong NIR absorbance could be used for PAI, and the high atomic number (*Z* = 73) of the W element could offer strong X‐ray attenuation ability for X‐ray CT imaging. Typically, a high CT/PAI contrast signal with remarkably enhanced CT value was achieved after injection (606.6 HU) than without injection (278.5 HU). The ability of simultaneous CT/PAI imaging with Cs*x*WO_3_ NRs@PEM could offer 3D whole‐body and high‐resolution images to guide precise cancer therapy in vivo (Figure 25D,E). Besides CT/PAI bimodal imaging, Cs*x*WO_3_ NRs@PEM could also generate both heat and ^1^O_2_ upon irradiation at either 1064 or 880 nm light. It was notable that both the temperature increment and ^1^O_2_ generation under 1064 nm irradiation were larger compared to that under 880 nm irradiation, which could help to penetrate deeper to treat deap‐seated tumors (Figure 25B,C). In vivo studies on HeLa tumor‐bearing mice following intratumoral injection of Cs*x*WO_3_ NRs@PEM showed remarkably increased CT and PAI signals, and the tumors shrank obviously after irradiation with 880 nm or 1064 nm light for 10 min (Figure 25F). Importantly, four and three out of five tumors were completely eliminated under irradiation with 1064 nm and 880 nm light, respectively, with no obvious side toxicity. These results suggested that Cs*x*WO_3_ NRs@PEM could act as an efficient theranostic agent for dual‐modal imaging‐guided combination PDT/PTT of tumors under irradiation with biological window NIR‐II light, which might be useful for the treatment of deep‐located tumors.

**Figure 26:**
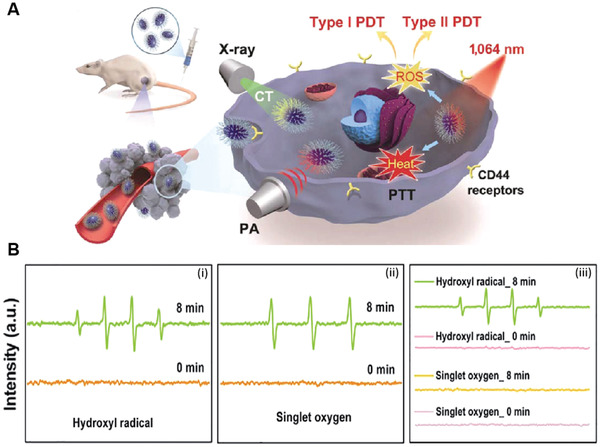
A) Schematic illustration of the application of HA‐W_2_C NPs for 1064 nm activated tumor dual‐type PDT, PTT, and PA/X‐ray CT dual‐modal bioimaging. B) ESR spectra of HA‐W_2_C NPs solutions before and after irradiation for 8 min: (i) and (ii) under normoxic conditions, and (iii) under hypoxic conditions. Reproduced with permission.^[^ ^122^ ^]^ Copyright 2018, Springer.

Instead of type‐II (^1^O_2_) ROS species, hyaluronic acid (HA) functionalized tungsten carbide (W_2_C) NPs developed by Yang's group showed both type‐I (·OH) and type‐II (^1^O_2_) ROS species, respectively, under normoxic conditions (**Figure** **26**A).^[^ ^122^ ^]^ Meanwhile, under hypoxic conditions, W_2_C NPs could also generate hydroxyl radicals as evidenced by EPR spectroscopy (Figure 26B) and in vitro DCFH assay. This illustrated that W_2_C NPs are suitable NPSs to treat the deep‐seated hypoxic tumor. Attributed to the high in vitro ROS generation capacity and photothermal conversion efficiency of W_2_C NPs, significant destruction of cells/tumors were achieved under 1064 nm laser (0.8 W cm^−2^) irradiation both in vitro and in vivo, respectively, without any obvious side effects. Apart from therapeutic performance, W_2_C NPs also showed enhanced CT/PA contrast images, enabling them to track cancer therapy in vivo. These findings suggested the promising potential of W_2_C NPs as a multimodal theranostic NPSs for cancer PDT of normoxic as well as hypoxic tumors. Importantly, in contrast to previously reported semiconductor‐based tungsten nanostructures, W_2_C NPs exhibited metallic character as revealed by diffuse reflectance spectrum and X‐ray photoelectron spectroscopy, respectively. Though both type‐I and type‐II ROS species were successfully detected, but their mechanism of generation remains elusive, which needs further exploration to advance the treatment of deep‐seated hypoxic tumors.

**Figure 27:**
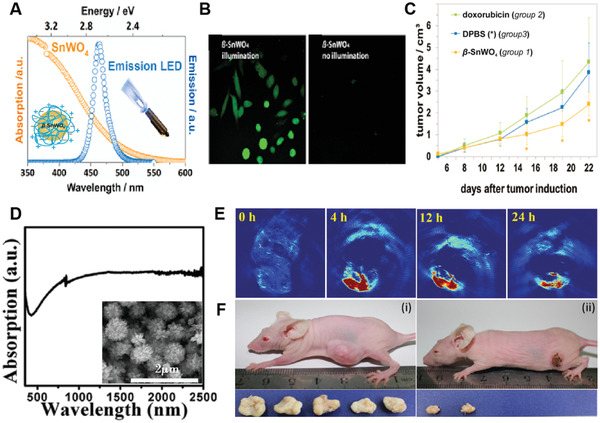
A) UV/vis spectrum showing the optical absorption as well as the emission of *β*‐SnWO_4_. B) Detection of ROS by *β*‐SnWO_4_ using fluorogenic H_2_DCFH‐DA with and without illumination of blue light LED. C) Effect of *β*‐SnWO_4_ NPs on the growth of the primary tumor in different groups. Reproduced with permission.^[^ ^123^ ^]^ Copyright 2016, American Chemical Society. D) Photoabsorption of WOs powder. Inset showed the SEM image of WO NPs. E) In vivo PA imaging of HeLa tumor‐bearing mice before and after injection of WO NPs for various times. F) Representative photographs of (i) control and (ii) treatment group (WO + NIR 1064 nm light irradiation) mice and tumors at 14th day. Reproduced with permission.^[^ ^125^ ^]^ Copyright 2017, Royal Society of Chemistry.

Apart from 1D tungsten‐based nanomaterials that have been widely used to treat deep‐seated tumors, Seidl et al. reported 0D tin tungstate (*β*‐SnWO_4_) NPs as inorganic NPS for PDT of surface located tumors under blue light illumination.^[^ ^123^ ^]^ In their work, *β*‐SnWO_4_ NPs with an average size of ≈8 nm were prepared, showing maximum absorption in the UV region with an emission at 470 nm, and further functionalized electrostatically with protamine to improve membrane penetration and cell uptake ability (**Figure** **27**A). The ^1^O_2_ generation efficiency of the *β*‐SnWO_4_ NPs in HeLa cells upon blue light illumination was evaluated by 2,7‐dichlorodihydrofluorescein diacetate (DHFA), showing a bright intracellular green fluorescence, which was not observed in HeLa cells treated with either *β*‐SnWO_4_ or blue light illumination alone (Figure 27B). Due to an efficient in vitro ^1^O_2_ generation, *β*‐SnWO_4_ NPs exhibited effective killing of both HepG2 and HeLa cells under blue LED light illumination for 5 min. Subsequently, the application of *β*‐SnWO_4_ NPs to trigger PDT of xenograft 4T1 breast tumors in living mice was conducted, which demonstrated remarkable tumor growth inhibition as compared to that of doxorubicin‐treated or untreated control mice (Figure 27C). Importantly, histological examination of lymph nodes revealed that the *β*‐SnWO_4_ NPs exhibited similar anti‐metastatic effect as that of doxorubicin, but at much‐reduced side effects as determined by blood counts. These results suggested that *β*‐SnWO_4_ NPs could be a new addition in already available inorganic NPSs, which will be much efficacious for the treatment of metastatic tumors and near‐surface PDT.

Meanwhile, in a recent work reported by Bu's group suggested the coupling of UCNPs with SnWO_4_ NPs to induce NIR‐triggered PDT and RT, offering dual‐modal therapy of deep‐seated tumors.^[^ ^124^ ^]^ Subsequently, to avoid the complexity of the system and extend the absorption of 0D tungsten NPs from UV to NIR region, Wang et al. prepared urchin‐like tungsten suboxide (WOs) NPs for photoacoustic (PA) imaging‐guided PDT/PTT in the second biological window.^[^ ^125^ ^]^ The designed WOs NPs showed broad absorption in the entire absorption spectrum (500–2500 nm) (Figure 27D), demonstrating significant heat and ^1^O_2_ generation under 1064 nm NIR laser irradiation, enabling effective HeLa tumor cell killing via PTT and PDT effects. The in vivo studies on HeLa tumor mice administered intratumorally with the WOs NPs showed remarkably enhanced PA signal (Figure 27E), and the tumors were completely eliminated after irradiation with a 1064 nm NIR laser (2 W cm^−2^) for 10 min (Figure 27F). Moreover, in vivo toxicological evaluation of intravenously injected WOs NPs did not reveal any noticeable systemic toxicity as monitored by hematological examination, indicating the favorable biocomptability of the designed WOs NPs. Thus, WOs NPs could serve as a simple but efficient multifunctional theranostic agents for imaging‐guide combination PDT/PTT of deep‐seated tumors.

## 9 Current Challenges and Future Prospects

As aforementioned, ^1^O_2_ generative nanomaterials are a rising platform that successfully demonstrated exciting results in PDT. In fact, nanomaterial‐based NPSs overcome and resolved most of the issues associated previously with classical organic PSs as well as showed remarkable therapeutic outcomes. Although these NPSs showed promising results owing to their highest ^1^O_2_ quantum yield, there are still many challenges that demand careful consideration for future clinical applications of these NPSs.

First, like organic PSs, the majority of currently reported NPSs, especially semiconductor NMs, are still activated under UV/vis light irradiation, which restricts their therapeutic applications to near‐surface tumors as well as peripheral and endoscopically accessible regions due to limited tissue penetration depth of UV/vis light. Currently, several strategies have been adopted, most notably decorating UCNPs onto semiconductors to tune their absorption from UV/vis to NIR region (biologically transparent window), which promises deeper tissue penetration depth and less attenuation by biological tissues. However, UCNPs based tunable absorption approach did not show satisfactory results due to certain limitations such as limited choices of excitation wavelengths (usually 980 nm), low extinction coefficient, and requirement of high laser intensity to generate upconversion visible light luminescence, which is far beyond the skin tolerance limit.^[^ ^126^ ^]^ Second, NPSs showed <10% accumulation in tumor region. To increase tumor enrichment, high dosage are usually administered intravenously, which can induce undesired side‐toxicity to the major organs. Warran Chan and coworkers recently demonstrated that nanoparticles extravasate into solid tumor through trans‐endothelial route rather than endothelial gaps. They suggested that by modulating the tumor endothelium, the enrichment of nanoparticles through trans‐endothelial pathway could be improved.^[^ ^127^ ^]^ Whereas, an in detail study should be conducted to elucidate how different tumor vessels and trans‐endothelial pathways respond to nanoparticles of varying diameter, morphology, and surface chemistry.^[^ ^128^ ^]^ Third, the clearance of these NPSs is an important question mark as they remained in the vital organs (e.g., liver, spleen, etc.) for longer periods after systemic administration and are nonbiodegradable, thus pose serious risk of long‐term biotoxicity. Yu et al. reported that rather than larger size Au NRs, smaller size Au NRs rapidly cleared from the body.^[^ ^129^ ^]^ Though these results are quite encouraging, it remains difficult to generalize which hydrodynamic size of NPSs is more appropriate for photodynamic tumor treatment in vivo as the nature of interaction between these NPSs and the biological systems is still unknown. In addition, in vivo biodistribution, long‐term toxicity, and biosafety profile of these rapidly developing NPSs are yet undefined, which is also a major hurdle in their bench to bedside applications.^[^ ^130^ ^]^ Furthermore, several factors such as size, surface chemistry, and inherent chemical constituents of nanomaterials are well known to modulate potential cytotoxic effects. For instance, CTAB‐functionalized Au NRs presented extensive cellular toxicity and low stability in physiological conditions. It has been found that CTAB as a surfactant disrupted the integrity of cell membrane by inducing multiple defects.^[^ ^131^ ^]^ Therefore, bicompatible surfactants should be considered for the stabilization of NPSs to ensure nontoxicity, biocompatibility, and good physiological stability. Recently, several studies have reported that many inorganic nanomaterials especially gold and carbon, if surface‐functionalized with a biocompatible agent (e.g., PEG) and an appropriate size, do not induce any noticeable toxic effect in cultured cells or mouse tumor models.

In addition to the above‐mentioned challenges, some future directions should be explored further to achieve high antitumor effects and to serve the suffering humanity. Since NIR laser can achieve deeper penetration within body, new NPSs that could be irradiated to directly generate a large amount of ^1^O_2_ at NIR‐I or even NIR‐II window should be developed to treat deep‐seated tumors in vivo. In particular, NIR‐II activatable NPSs would not only resolve an issue of low penetration depth but also reduce the overall complexity and cost of the system.^[^ ^132^ ^]^ Recently, Huang et al. demonstrated an innovative strategy to modulate the plasmonic peak of gold‐based theranostic systems in NIR‐II region to achieve deep‐seated tumor theranostics.^[^ ^133^ ^]^ Meanwhile, biocompatible and biodegradable NPSs (e.g., black phosphorus NSs, hybrid‐based NPSs) capable of reducing side toxicity in vivo are greatly needed.^[^ ^134^ ^]^ A recent report from Wang's group demonstrated a combination of nanomaterials both as exciting moiety and as NPS. Plasmonic Au NRs were used as a primary exciting moiety to activate the attached carbon dots‐based NPSs due to the near field enhancement effect, showing high ^1^O_2_ generation under NIR light as reported from GQDs alone.^[^ ^92^ ^]^ In our opinion, this is the next generation of PDT, which used nanomaterial to excite the attached NPS. This new direction not only overcomes most of the issues of traditional PDT based on organic PS but also paves the way for the development of multifunctional nanotheranostic agents exclusively based on nanomaterials. Moreover, this approach should be expanded to other nanomaterials by taking careful considerations of the spectral overlap of the maximum absorption wavelength of both the nanomaterials. Fourth, Zhang et al. first time reported the in vivo high PDT efficacy based on dual organic PSs coupled with UCNPs.^[^ ^135^ ^]^ Very recently, Hou et al. use a combination of organic PS (Hypocrellin A) and inorganic PS (TiO_2_ NPs) by integrating with UCNPs, while Feng et al. utilized nanomaterial‐based dual inorganic NPSs (g‐C_3_N_4_ and Au_25_ nanoclusters) coupled with UCNPs and evaluated their in vivo anti‐tumor performance.^[^ ^119^ ^]^ Both observations demonstrated the high PDT response and better tumor inhibition compared to single PS. Hence, this approach should be explored further to understand how dual NPSs could be used to enhance the ^1^O_2_ quantum yield, which is of course indirectly improve the treatment outcomes. Furthermore, the utilization of dual NPSs having maximum absorption at NIR and UV/vis region will provide an edge to the scientific community to get the ultimate therapeutic results by taking the advantage of an entire light spectrum (200–1000 nm) capable of treating the near surface and deep‐seated tumors, simultaneously. As we all know that single treatment modality is not sufficient to completely eradicate the tumor and there is a consensus among scientific community regarding this issue. Therefore, new NPSs should be developed, which will enable simultaneous PDT and PTT. Recently, Vankayala et al. and Wang et al. reported Au NRs and CuS nanocrystals as both PDT and PTT agents.^[^ ^37^, ^74^ ^]^ Therefore, it is highly demanding that multifunctional nanoagents with multimodal imaging and synergistic therapy should be developed to win this battle against cancer. Metal‐organic frameworks (MOFs) are new and exciting material, showing remarkable contribution in PDT as a nanocarrier due to their certain inherent features such as adjustable pore sizes, crystalline nature, design flexibility, tunable chemical environment, biocompatibility, high porosity, and high surface area. Recently, MOFs as a nanocarrier, have been successfully integrated with organic PSs and exhibited satisfactory therapeutic performance.^[^ ^136^ ^]^ Recently, Lu et al. and Shang et al. reported nanomaterials encapsulation within MOFs and then further applied them in cancer diagnosis.^[^ ^137^ ^]^ However, there is no such report to date, which demonstrated the integration of NPSs within MOFs for PDT. In fact, nanomaterials encapsulation within MOFs is highly beneficial regarding PDT, because MOFs will provide high payload as well as avoid premature release of entrapped nanomaterials, and hence ensure better therapeutic results. Thus, there is much room in this area that seriously needs attention, and ultimately will be quite beneficial for the designing and development of next‐generation multifunctional nanotherapeutic agents. Besides these advancements, it is of great significance to conduct nanotoxicological studies of these rapidly emerging NPSs by considering different parameters, for example, surface chemistry, hydrodynamic diameter, biodistribution, biodegradation, etc. to assess their potential long‐term safety concerns. Currently, Au nanoshells showed preliminary success in the clinical trials,^[^ ^17^ ^]^ which would truly shine some light on the toxicological evaluation of nanomaterials, leading to the development of biodegradable and biocompatible nanotherapeutic agents for PDT/PTT with significantly higher clinical value. A biological risk assessment is also mandatory for their transformation into clinic, and also for designing and developing personalized cancer inorganic nanomedicine. In addition, though few reports suggested deep‐seated tumor treatment by NIR‐II activatable NPSs, they are usually based on subcutaneous tumor mice model. Since several tumors are usually presented in deeper tissues, large animal models with deep‐seated tumors should be developed for in‐depth rigorous investigation of these NIR‐II based NPSs to ascertain their practical clinical applications to treat deep‐seated tumors.

In summary, the last decade has witnessed remarkable advancements in the development of ^1^O_2_ generative nanomaterials because of their tremendous applications in PDT. Nanomaterial‐based NPSs provided the highest ^1^O_2_ quantum yield and exhibited promising potential as modern PSs compared to conventional organic PSs. Owing to their enormous advantages, this new class of PSs would have the potential to compete and replace organic PSs to achieve high PDT efficacy. It is envisioned that NPSs will provide improved selectivity, reduced cytotoxicity, and excellent tumor targeting capacity and hence will show profound effects on cancer PDT. With the rapid development of nanotechnology, it is expected that new nanomaterial‐based NPSs with multifunctional properties will be developed in near future, which will really make dramatic breakthrough discoveries in cancer therapy and ultimately shift this treatment approach from bench to bedside. We are quite hopeful that this review provides a timely and extensive overview of the current status of this new dimension and will truly shine some light on the outlook of future research of PDT to move forward and conquer this battle against cancer.

## Conflict of Interest

The authors declare no conflict of interest.
